# Supplementary material for: The detection of algebraic auditory structures emerges with self-supervised learning
Source: PLoS Comput Biol. 2025 Sep 5;21(9):e1013271. doi: 10.1371/journal.pcbi.1013271 (PMC12431648; doi:10.1371/journal.pcbi.1013271)
Supplement: S1 Material — (PDF) [file pcbi.1013271.s001.pdf]

Supplementary Note 2: Supplementary results

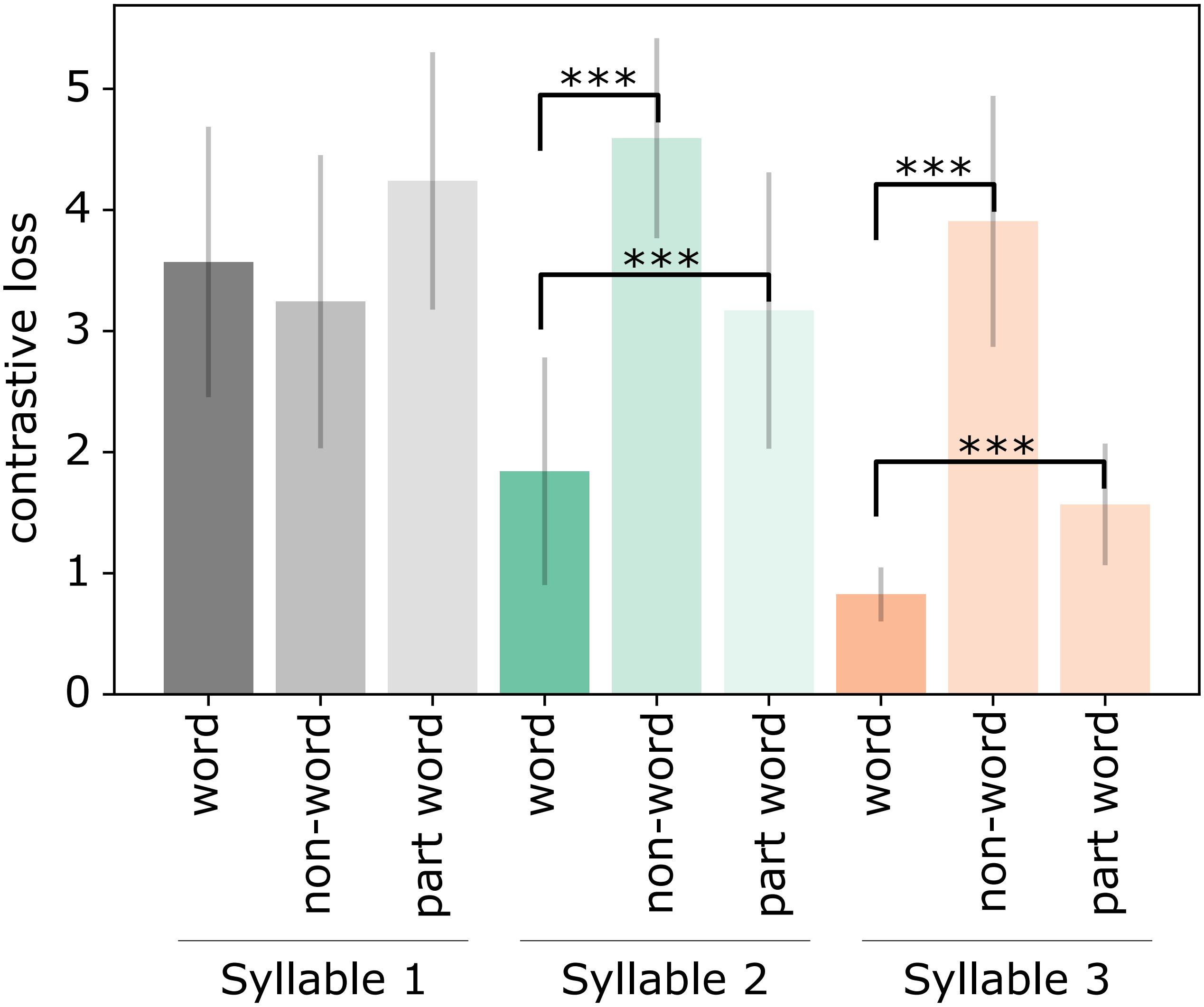

Supplementary Figure A. Contrastive loss to seen word, non-word never seen and part-word (seen). Left three bars plot the average contrastive loss for the first syllable. The middle three bars plot the average contrastive loss for the second syllable. The right three bars plot the average contrastive loss for the third syllable. Error bars capture the standard deviation across 30 trials. If the word is detected, the surprise, i.e. contrastive loss, should be larger at syllables 2 and 3 for the non-word seen and even smaller for the non-word never seen compared to the true word. This is indeed the case for syllables 2 and 3 (t-tests, p-value<0.05)

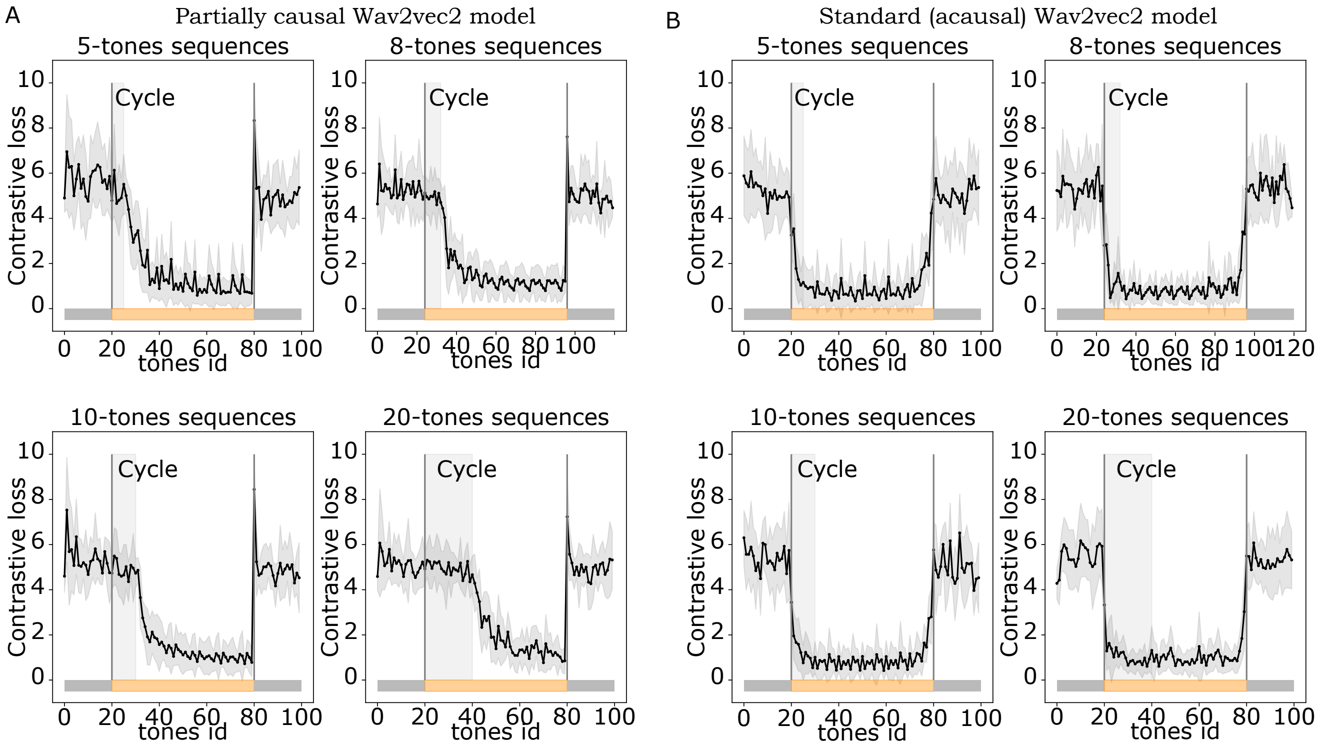

Supplementary Figure B. Contrastive loss of a causal (left) and acausal (right) models in responses to N-tones sequences ($N \in \{5, 8, 10, 20\}$). The acausal model detects the regularity earlier than the causal model.

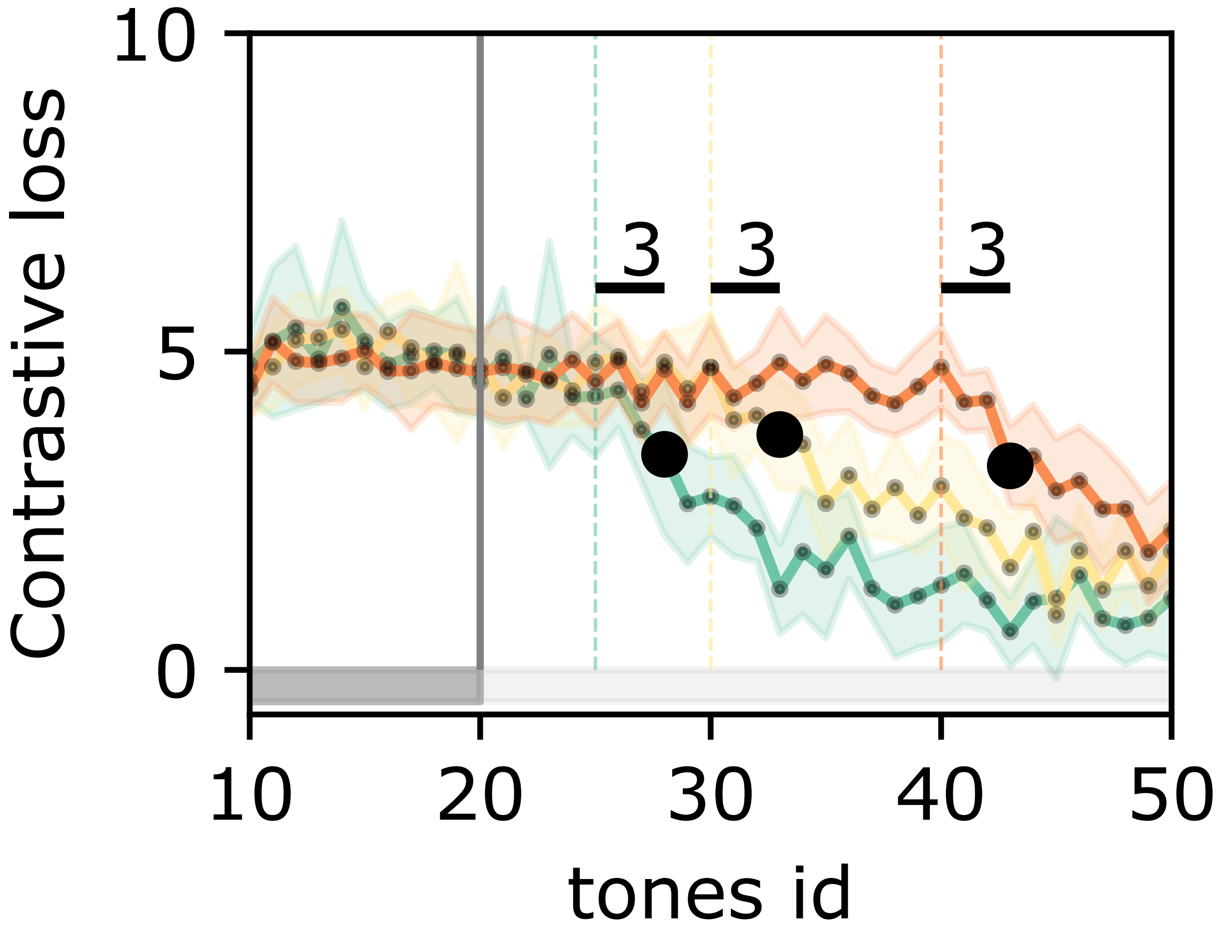

734

Supplementary Figure C. Zoom on the transition from random to regular. We mark as black dots the first tones whose contrastive loss was significantly different from the contrastive loss of the final set of random tones. We indicate in black the distance between this tone and the end of the first cycle. The significance is assessed with t-test for independent samples across the 30 trials, and we choose for p-value a threshold of 0.005. Note that this result is dependent on the p-value threshold chosen, such that we would not qualify it as a robust result. Nevertheless, the model is in the order of magnitude of the number of tones at which the detection should happen as predicted by an optimal Bayesian observer in (23), and at which humans detect the tones.

Test Stimulus	Model	Studies			
		AlRoumi2023	Barascud2016	Saffran1996	Bekinschtein2009
natural sounds	environmental pretraining	2.50	5.45	4.43	1.58
	music pretraining	7.41	5.88	3.83	6.58
	**speech pretraining**	**0.16**	**0.48**	**0.02**	**-0.02**
speech (syllables)	environmental pretraining	0.2	1.96	1.92	0.10
	music pretraining	0.23	2.39	2.26	0.97
	**speech pretraining**	**-0.04**	**0.17**	**-0.06**	**-0.10**
tones	environmental pretraining	0.76	1.45	1.69	1.68
	music pretraining	4.68	4.50	2.9	4.17
	**speech pretraining**	**-0.02**	**0.27**	**-0.26**	**-0.19**

Table A. Cross dataset evaluation: We evaluate each model on the 4 types of sequences changing the sequence elements to environmental sound, syllables, or tones. In all situations, speech models are never able to detect the auditory structure. This shows that the difference between the models is not explained by a difference in the type of test elements.

Model Type	Model	References			
		AlRoumi2023	Barascud2016	Saffran1996	Bekinschtein2009
wav2vec2_tiny	music tiny	4.16	3.22	2.72	2.96
	speech tiny	-1.17	0.06	-0.44	-0.58
wav2vec2	music	4.68	4.50	2.90	4.17
	speech	-0.02	0.27	-0.26	-0.19

Table B. Differences between music and speech models are robusts to a change in the model architecture.

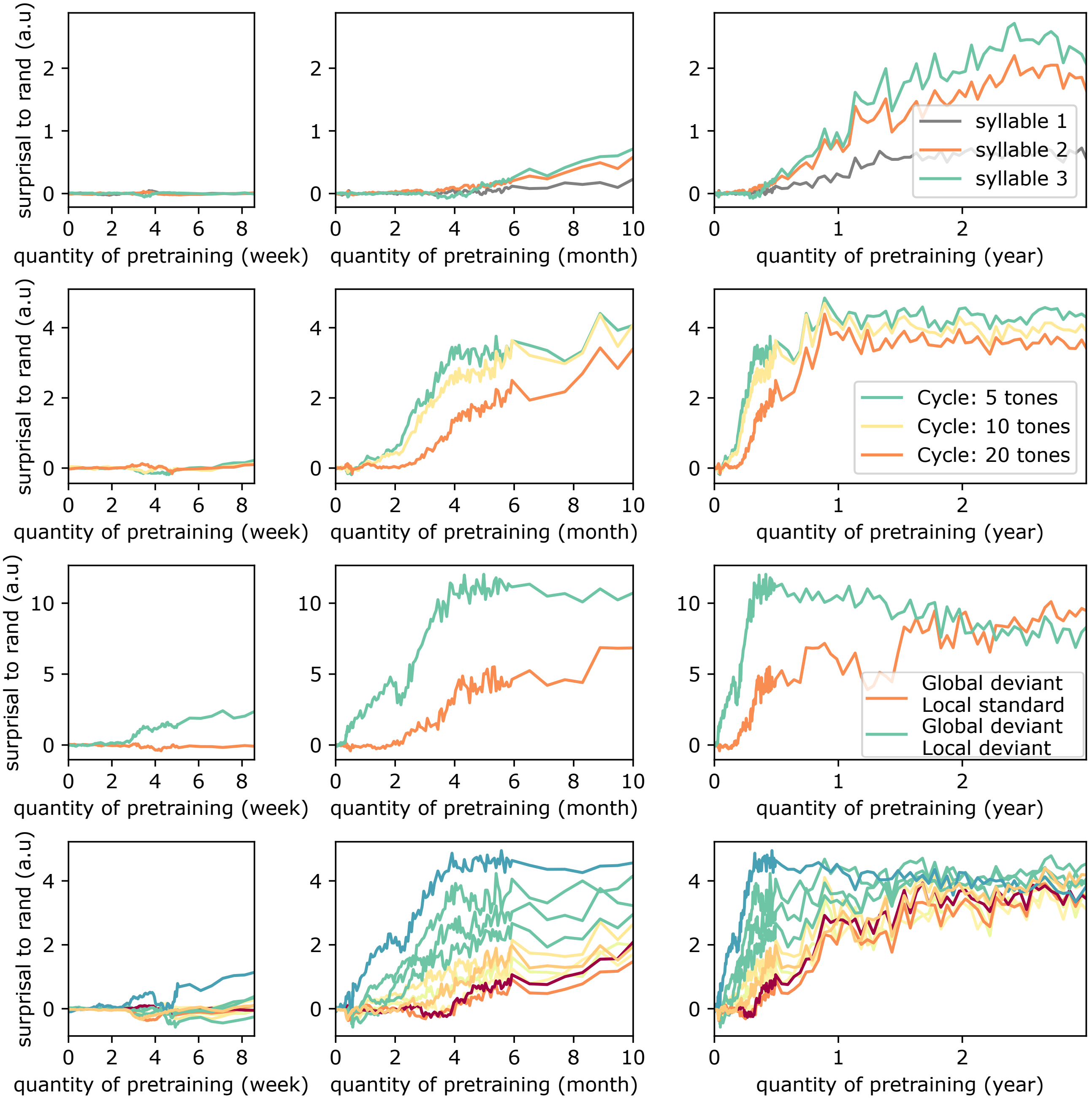

Supplementary Figure D. Zoom on Fig 2, for each of the four experiments. First row: note the late emergence of syllable chunking, starting between 6 and 8 months. Second row: note the 2-months delayed emergence for the longer 20-cycle sequences compared to sequences of cycles 5 and 10. Third row: note the later emergence of Global deviant - Local standard detection compared to Global deviant - Local deviant. Fourth row: note the sequential emergence as a function of the sequences' complexity between 2 and 6 months.

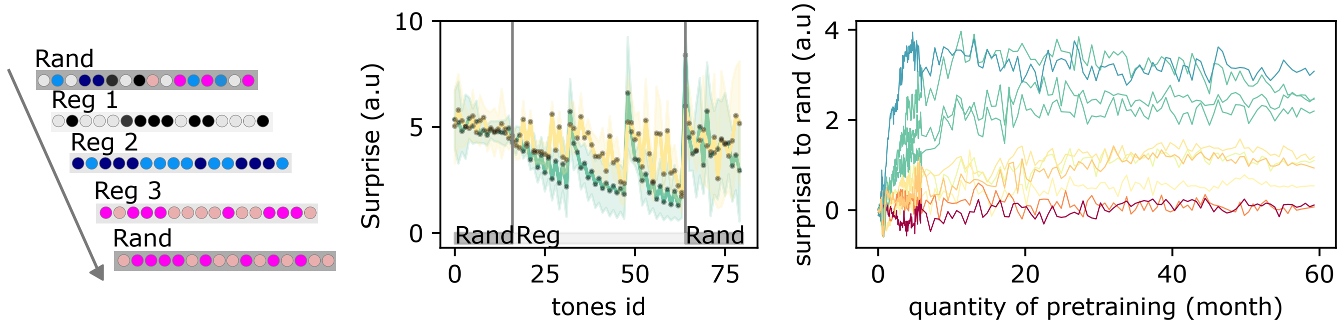

Supplementary Figure E. Generalization protocol. We test if the networks generalize to sequences with novel sound elements but shared structure. Sequences are the same as before, i.e, the algebraic patterns of (8). At the onset of each repetition of the structure, the loss peaks because the model doesn't know the novel elements. In the third and second repetition, as the model detects that these novel elements follow the same structure, the loss quickly decreases, to finally reach a lower surprise than the preceding sequences, and demonstrate a strong surprise to a random novel sequence. We measure on the right, the difference between the loss over the last 10 tones of the third sequence and the random pattern. Indeed we can't use the loss over the last 16 tones, as we need to let the model see at least 2 tones, which happens at the 5th tones for some sequences, forcing the use of the last 10 tones only.

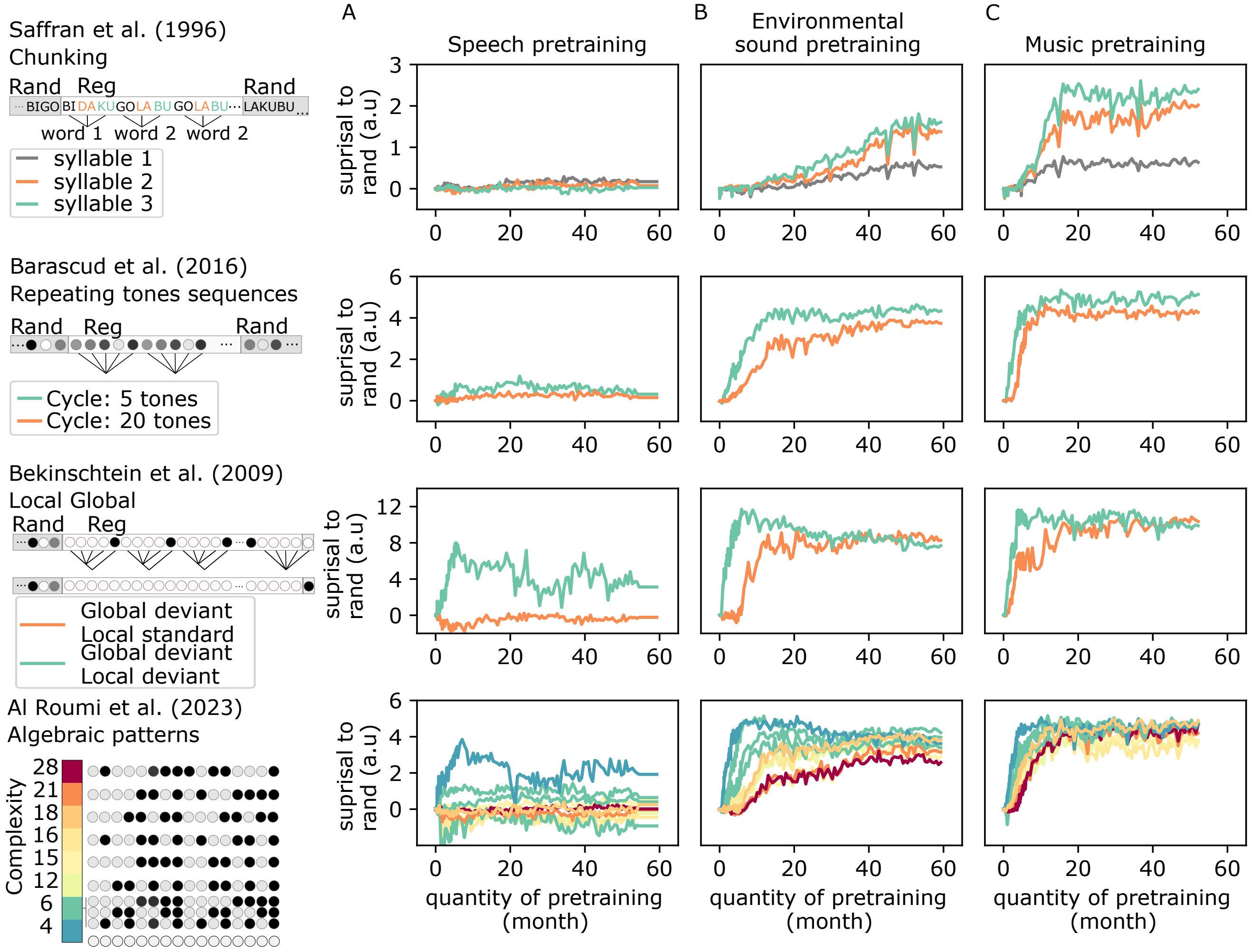

737

Supplementary Figure F. Same as Fig 3, but evaluated on a second set of pretrained models. We observe the same dynamics as in the original figure, indicating that our results are robust to the random initialization of the models before pretraining.

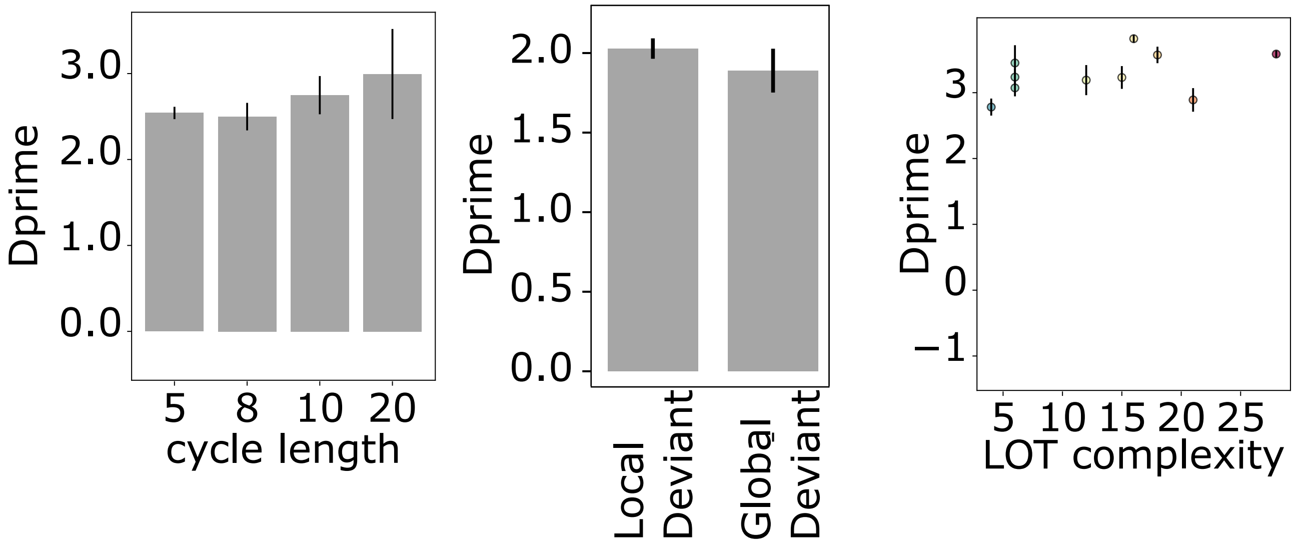

738

Supplementary Figure G. Ability of the model to detect single deviant in sequences of tones. The model can detect (high D prime) deviant tones across all types of sequences.

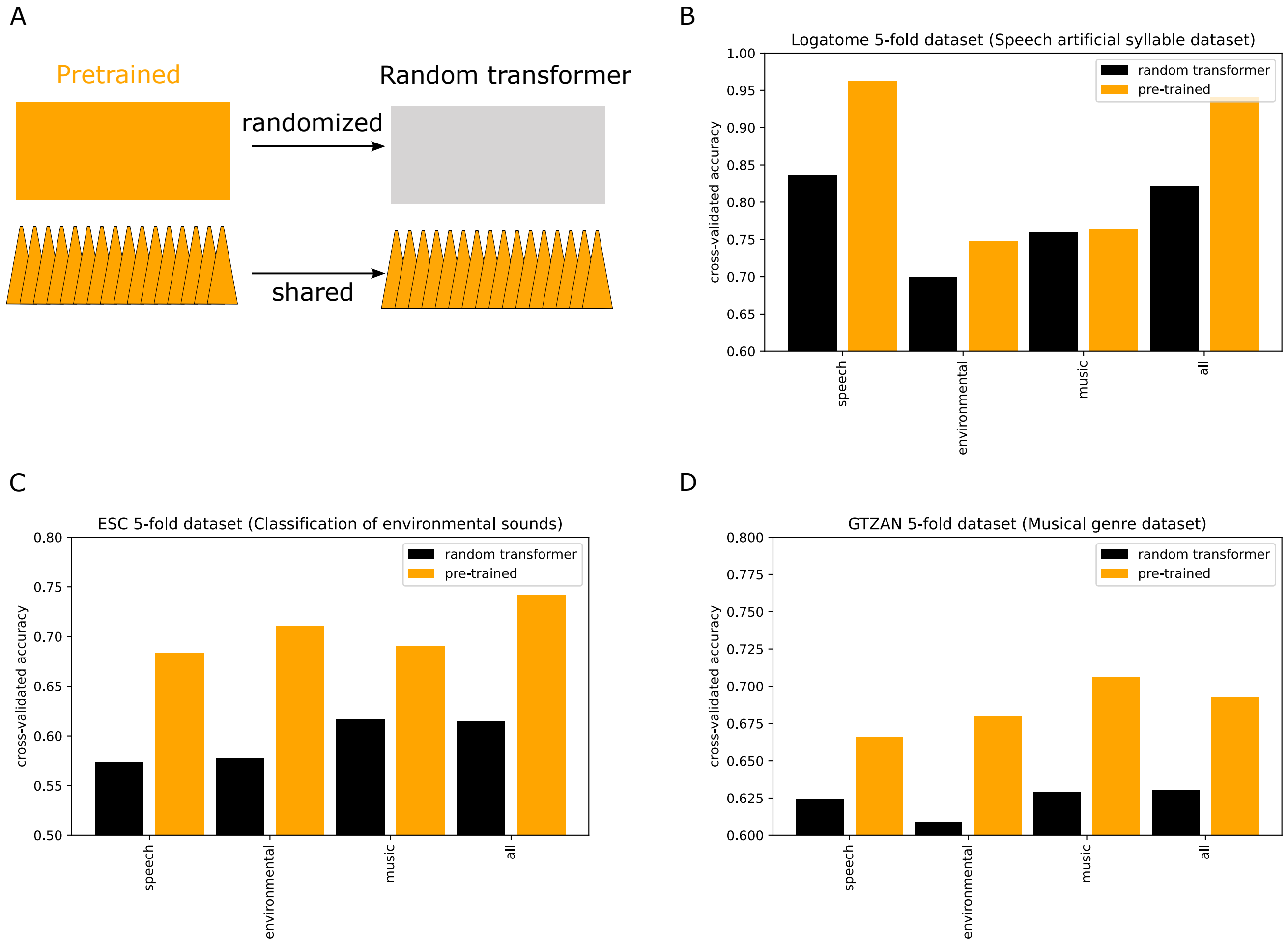

Supplementary Figure H. A. We evaluate the pretrained models' representations in two conditions. Models were either unchanged (left) or their transformer layers randomized while preserving the learned parameters of their convolutions layer (right). B: Classification performances on logatomes. C: Classification performances on environmental sounds. D: Classification performances on musical genres. Comparing orange and black bars allows us to perceive the added benefit of contextual integration versus the purely local filtering of the convolutions. The black bars are not to be taken as a fully random baseline, which would require randomizing the convolutions as well.

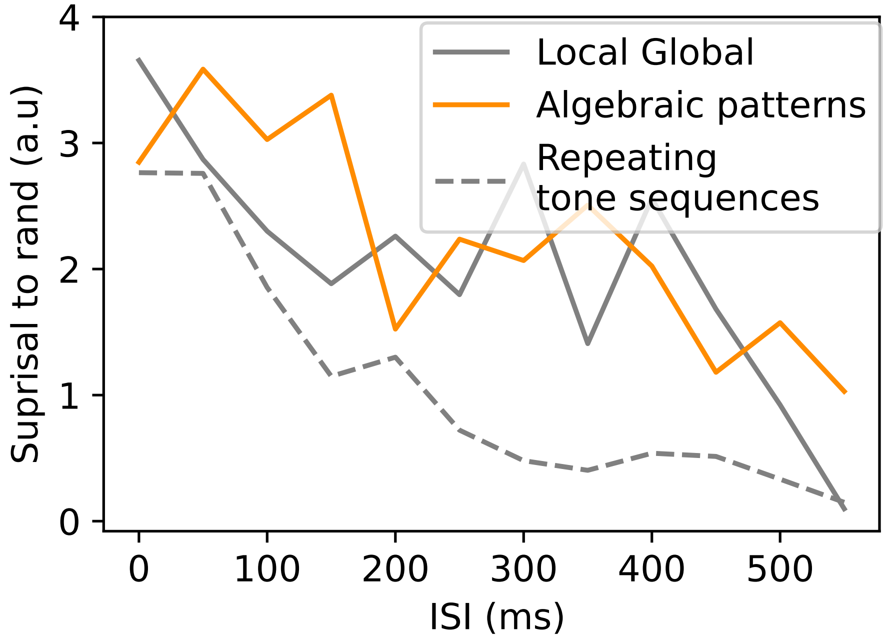

Supplementary Figure I. Decrease of the one-shot detection ability at large inter-stimuli interval (ISI). We measured the loss gap between random and regular sequences as a function of the silence duration between tones used for the binary-tones and N-tones experiments.

A. Structure with longer inter-stimulus intervals (ISI) are harder to detect. We questioned if the model was robust to the insertion of silences in the sounds. We therefore added a constant inter-stimuli silence interval (ISI) between elements of the repeating tone sequences and algebraic sequences. We then repeated our evaluation with the model trained on all sound types. Problematically, we observed that the model started to diverge from its expected behavior at large ISI. For example, in the presence of silence, the model's surprise to a repeating tone increased with the number of repetitions, whereas it should stay low near 0. We hypothesized that this effect was due to the normalization occurring on the waveform and after the first layer of convolution. Indeed, in the presence of a repeated element or repeated silences, this normalization biases the extracted local representation, which in turn impairs contextual processing. Removing the waveform normalization helped mitigate this issue but the problem remained at longer ISI due to the normalization in the second convolutional layer (Fig I in S1 Material). Removing or changing this normalization to be computed per non-silenced sound bouts did not improve the model behavior. This issue will therefore have to be resolved by a change in the model architecture and novel pretraining.

Nevertheless, in ISI ranges typically used in experiments tackled here (200ms for (8), 150ms for (21)), the model has a reasonable behavior (Fig I in S1 Material). The model performed robustly to additional silences of 0.5 or 0.75 seconds between algebraic sequences. For repeating tone sequences, the slight decrease of performance with silence of 200ms mirrored the recent results of (56), where participants performed the same task as in Barascud et al. (23) but with increased difficulty as the silence gap increased.

Overall, we conclude that the model lacks robustness to silences, especially because of two successive normalizations. Removing the first operation of normalization helps, and in this regime, the model detects structures at experimental ISI ranges. In

the regime of over-normalization, the model abilities collapse for silences beyond 200ms. We therefore conduct further explorations, reported in detail below, questioning if the model abilities can resurface through a few-shot scenario. In this scenario, the model is allowed to learn from backpropagation and tested on deviant sequences. The model ability was recovered after a small (<50) number of backpropagation steps. We therefore hypothesize that further improvement of the models, for example by forcing silences to be present during pretraining, could be sufficient for the model to be robust at all ISI.

B. Learning from few-short exposure to regular structures.

Can online self-supervised learning improve the detection of repeated structures? In all of the above experiments, the model was evaluated in "zero-shot", meaning that test stimuli did not change any of the internal parameters of the model. While zero-shot detection cannot trivially depend on synaptic plasticity, repeated exposure to the test sequences unfolds over long time periods that allow synaptic plasticity to change the network. Therefore, we tested a more permissive – yet plausible – "few-shot" scenario, where the model was plastic and could change its internal parameters upon successive presentations of the repeated structure. Specifically, we mirrored human experiments (8, 23), and repeated the standard sequences over several seconds to minutes. The model could adapt to the test stimuli through several gradient descent steps (Fig J in S1 Material), using the same unmasking objective used for pretraining. We tested whether this few-shot exposure led to enhanced extraction of standard sequences and algebraic structures by evaluating the contrastive loss on the deviant sequences. To face the model with challenging stimuli, we used ISIs of 250 ms. To track the learning of the models over stimulus repetitions, we tested the model every 5 checkpoints from step 5 to 50, and then every 50 steps up to 600.

Repeating tone sequences We first tested the effect of few-shot exposure with repeating tone sequences at an ISI of 250 ms. The habituation was composed of four repetitions of the tone motif. In the test sequence, the fourth sequence was changed by swapping one tone for a deviant tone (Fig K in S1 Material). The model detected repeated structures in the zero-shot scenario but improved with repetitions of the standard sequence (Fig K in S1 Material).

Global deviant - local standard Next, we habituated the model to the XXXXY sequence and tested its ability to be surprised by a global deviant - local standard token in last position (XXXXX) (Fig K in S1 Material), with an ISI of 250 ms. Habituation sequences were composed of three repetitions of the local deviant sequence: XXXXY. The test sequence was composed of two repetitions of XXXXY followed by the global deviant - local standard XXXXX. The ability to be surprised by the global deviant - local standard emerged (Fig K in S1 Material) but only when the model was exposed at least 35 times to the sequence. We repeated the experiments for different learning rate parameters and across the eight fully-pretrained models, but none were able to detect the global deviant in less than 20 steps of gradient descent (Fig L in S1 Material).

Algebraic patterns We tested the model's ability to memorize algebraic patterns by measuring its sensitivity to 4 alternative deviants per sequence, as used in (8) (Fig K in S1 Material). The model was able to memorize all sequences, but simpler sequences were memorized faster than more complex sequences (Fig K in S1 Material). As humans, after being exposed to 10 repetitions, the model sensitivity was correlated with sequence complexity ($R = -0.86 \pm 0.05$, statistics across the last 10 pretraining checkpoints). We performed several controls to further explore this finding. First, the memorization of each

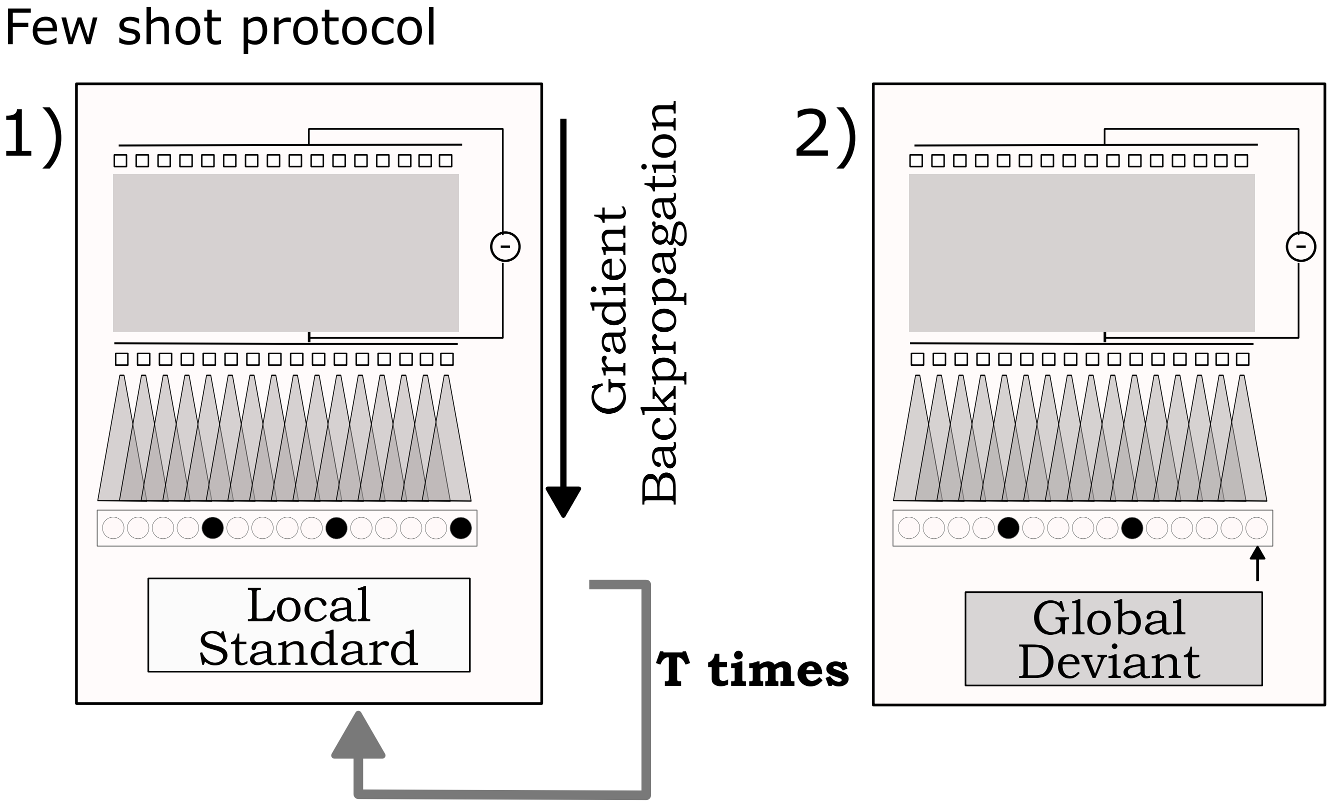

Supplementary Figure J. Few-shot protocol. 1) A sequence (here the Local Standard of the Local Global paradigm) is presented repeatedly to the model. Between each presentation, the model parameters are updated through backpropagation. 2) The model surprise on a deviant sequence is then measured (here the Global Deviant of the Local Global paradigm).

sequence could depend on their duration, which largely differs (Figure 1A). Sequences with smaller cycles are more repeated and therefore could be learned faster, which would explain the correlation between sequence complexity and learning speed. To test this hypothesis, we generated 10 novel sequences which were all of duration 16 tokens, but varied complexity. Model sensitivity stayed high (min D'=1.57, max D'=4.0, mean D'=3.56) and still correlated with sequence complexity (R = -0.66, Fig M in S1 Material). Second, we tested if the model could generalize structure detection to novel sequences, based on the same algebraic pattern but different sound elements. We replaced the repeated exposure to standards with the repeated exposure to 3 novel sequences, each composed of different sound elements while preserving a shared algebraic pattern. We tested the model on a fourth sequence, also composed of novel sound elements but the same shared algebraic pattern. The model generalized from all but the most complex pattern, with other nested patterns requiring at least 50 repeated exposures (Fig N in S1 Material). Finally, we stress that despite the model's ability to perform few-shot detection at long ISI, the model behavior and dynamic of emergence are much noisier in this few-shot scenario than during the one-shot scenario at low ISI (Figs L, M,N in S1 Material). Therefore, such models have to be improved to exhibit structure memorization as robustly as humans.

Distinguishing diverse complexity metrics To better understand the model behavior, one can measure if it correlates with known complexity metrics, as each indicates the sort of compression algorithm the model could be performing. We follow Planton et al. (82) and Al Roumi et al's method (8) and investigate model correlation with several alternative complexity metrics. The complexity metrics are defined extensively in (82) and summarized here:

- LoT Complexity: The length of the shortest description of a sequence, using a specific formal language with two instructions, "same" and "change".
- LoT Chunk Complexity: This metric is a variant of LoT complexity in which the shortest description is constrained by respecting the chunks of consecutive repetitions and never splitting any such chunk.
- Chunk Complexity: This metric is based on a formula that takes into account the number of chunks in a sequence and the length of each chunk
- Entropy: This metric quantifies the uncertainty of the transition probability distribution.
- Lempel-Ziv Complexity: This metric derives from a lossless data compression algorithm. It works by scanning a sequence and adding new substrings to a vocabulary, with the final complexity being the number of substrings in the vocabulary.
- Number of Subsymmetries: This is simply the number of symmetrical subsequences of any length within a sequence.

We observe a modest correlation between the detection scores (D') of the wav2vec 2.0 model and the sequence LOTC O (R=-0.52+/-0.13). This correlation is measured every 50 exposition steps, but peaks early on at R = -0.86 ± 0.05, if only 10 exposition steps are used. Overall, this analysis suggests that the model behavior correlates mostly with a particular type of complexity metric, defined through a simple recursive algorithm.

Methods for the few-shot protocol. To test if the network is able to learn sequence structure if we turned on plasticity, we pursued its pre-training by performing 600 gradient descent steps using the exposed sound. As for pre-training, we used the HugginFace Trainer implementation (75), with the same modification to the Wav2vec2 implementation of HuggingFace as for

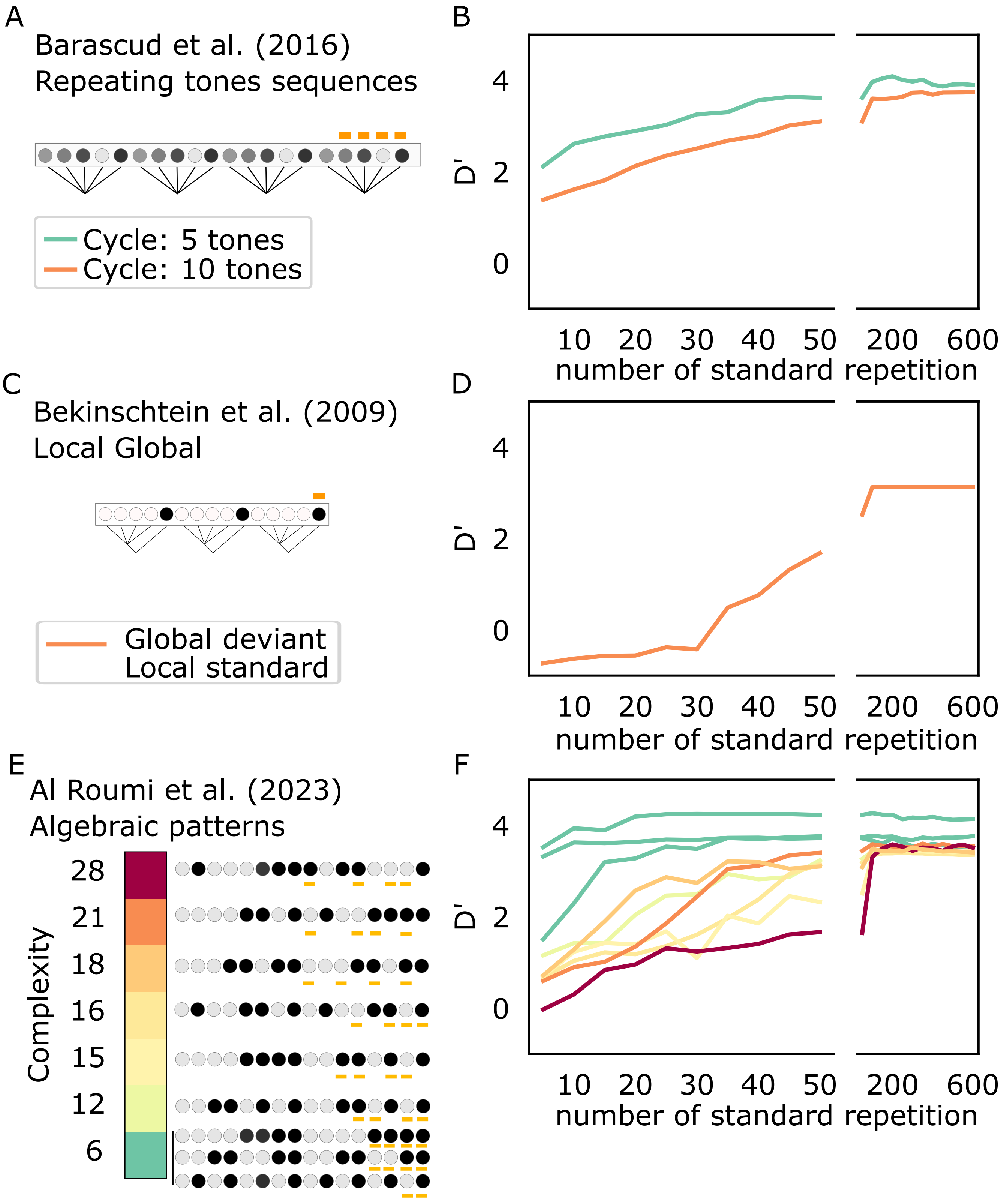

Supplementary Figure K. Self-supervised gradient descent led to sequence memorization modulated by structure complexity. A: The model was exposed repeatedly to four repetitions of a random sequence of 5 or 10 tones, with 250 ms inter-tone interval. The model was then tested on deviant sequences, in which one tone (marked by orange ticks) was swapped for another deviant tone. B: Model sensitivity to deviants as a function of the number of exposition. C-D: Same as A-B but for the local global paradigm of (21). E-F: Same as A-B but for algebraic patterns of (8).

pre-training (replicating the original Wav2vec2 implementation available in FairSeq). The Adam optimizer used during pre-training implements a learning rate scheduler, leading to small weight modifications at the end of pre-training. To maximize the effects of the exposition, we therefore re-initialize the optimizer for each of these tests. The ADAM optimizer learning rate decreased with a linear ramping from a maximal value to 0 across these 600 gradient descent steps. We explore several learning rate maximal value: $2.5 * \times 0^{-4}, 0.759^{-4}, 2.59^{-5}, 0.759^{-6}, 2.59^{-6}$. Results were consistent across learning rates, except at the largest one where no convergence was observed. We report results for a learning rate of 2.59^{-5} in the main paper, and results for all learning rates can be observed in the supplementary.

Across steps, we first iterate through the different masks, with each mask covering one of the sound elements present in the sequence, and then through 3 repetitions of the same sequence (detection set-up) or through concatenation of 3 sequences with shared structure but different tones. These 3 sequences mirror the "zero-shot" scenario where they were concatenated one after the other. At test time, we use either the same sequence (detect) or a novel sequence with the same structure but novel sound elements (generalize). The optimization is performed with a batch of size 16 and uses a single GPU. A batch of size 16 allowed to perform gradient descent simultaneously through all the masks corresponding to the 16 tones of the binary tone sequences.

During these gradient descent steps, the model employs the same regularization tool as during pre-training, notably unit dropout and layer dropout. We observed that a dropout of the first layer generated loss spikes, which disappeared when layer dropout was not allowed. Although this could participate in the fragility of the model in the few-shot scenario, we preserved this dropout to mirror, in few-shot, the updates done in pretraining, and, more generally, stick to the hyperparameters found by the original author of the Wav2vec2 model (41).

Finally, note that the HuggingFace Trainer uses a gradient scaler for technical purposes. We observed that this scaler led to the skipping of the first few steps of optimization to find an adequate gradient scaling factor, notably because we re-initialized the optimizer. We recorded which of those steps were missing, and accounted for them such that step five reported here was exactly the fifth step of gradient descent. These variations have therefore no impact on the results we report here.

D' measurement. The D' is estimated from the model loss to all sound elements of four different deviant sequences. For each of these deviant sequences, a different sound element is replaced with another from the sequence. For example, one tone is replaced with its binary alternative in binary sequences. For a sequence of size N, a threshold on the loss then determines which elements are classified as predicted or not predicted by the model. To have high sensitivity, every element among the N elements of the sequence should be classified as predicted, except the deviant which should not be well predicted (i.e. deviants should have a larger loss than other elements). The threshold determines which elements are predicted versus non-predicted. We compute the sensitivity of this particular classification by merging the results across the 4 deviant sequences and computing a D' from this set of 4N classifications. This operation is repeated for all of the 4N possible threshold values. More precisely, we change the classification boundary so that one element changes class at a time, starting from the one with the largest contrastive loss to the one with the smallest contrastive loss. We report the D' measure of the model sensitivity on this sequence as the average of these 4N measures, weighted by the 4N loss gaps between each threshold change. This weighting procedure allows us to take into account how far the loss was on the deviants compared to other elements. We observed manually that this

measurement captured well the model's behavior.

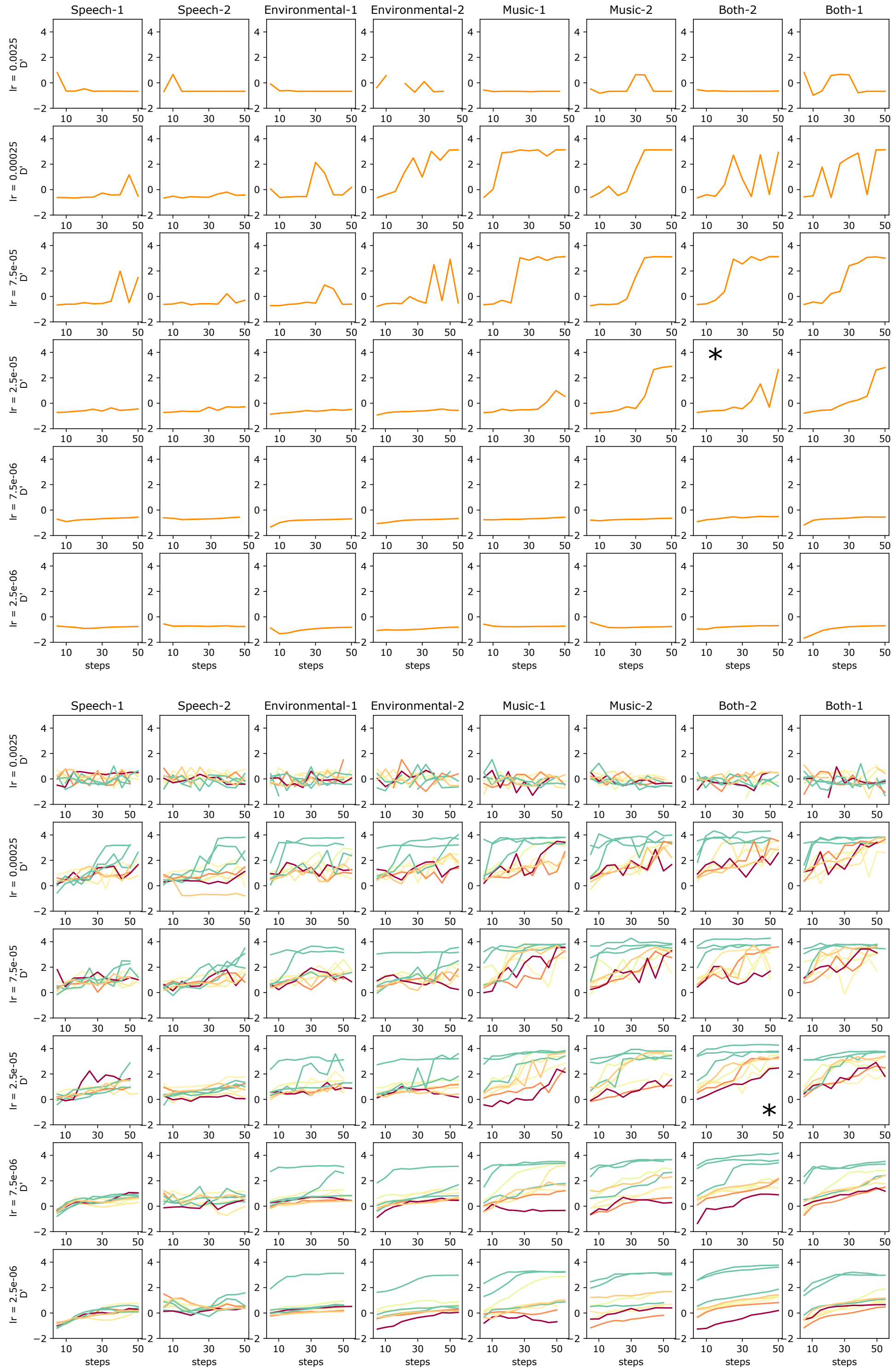

Supplementary Figure L. Structure detection in the few-shot scenario, across learning rates (lr) and models. Top: sensitivity to the Global deviant - local standard of the local-global paradigm of (21). Note how the performance improves with the learning rate but still requires more than 20 steps of learning. The star indicates the model and learning rate plotted in the Fig K in S1 Material, with the difference that in Fig K in S1 Material, the operation was repeated for the last 10 pretraining checkpoints and the d' averaged across this checkpoint. Here, we only measure and report the d' for the final checkpoint. Bottom: sensitivity to the deviants in the algebraic pattern paradigms of (8). Note that these evaluations are performed only once per sequence. The fragility and randomness of the model behavior in the few-shot scenario are revealed by the non-monotonous aspect of the curves.

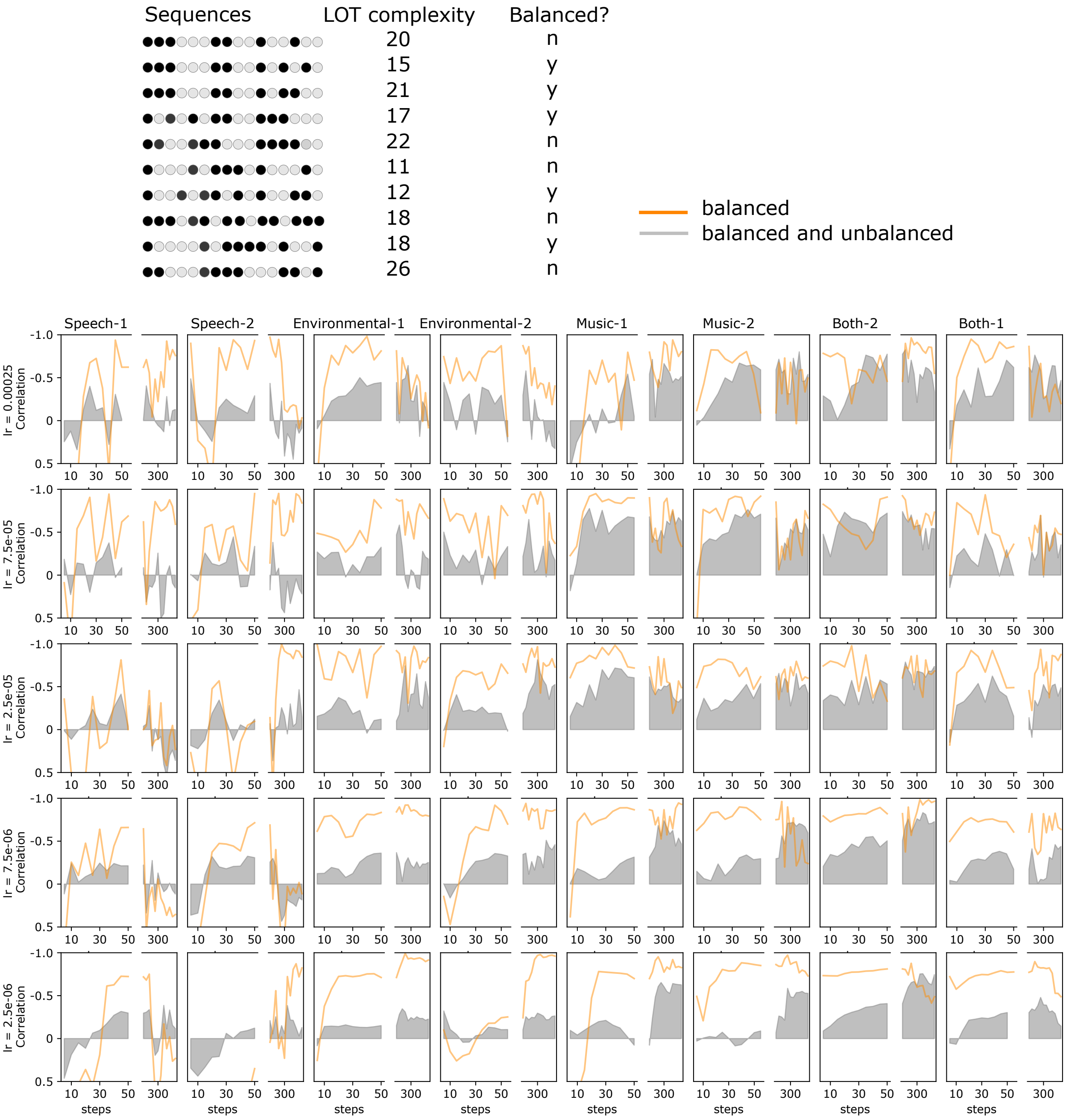

Supplementary Figure M. Correlations of the LOT complexity and the model sensitivity for 10 novel sequences of cycle 16 but varied complexity, either balanced as in algebraic patterns of (8), or imbalanced as in the local global paradigm. We plot in grey the correlations of the surprise to all sequences and LOT complexity and in orange the correlation of the surprise of the 5 balanced sequences and LOT complexity. This correlation is plotted as a function of the habituation steps. The model is modestly correlated with the LOT complexity for most models except speech models where the correlation remains low. This correlation is further improved by using only balanced sequences in most cases. Once more, we note that in this few-shot scenario, the model abilities are fragile: they vary with the learning rate and are non-monotonously increasing with the steps of habituation.

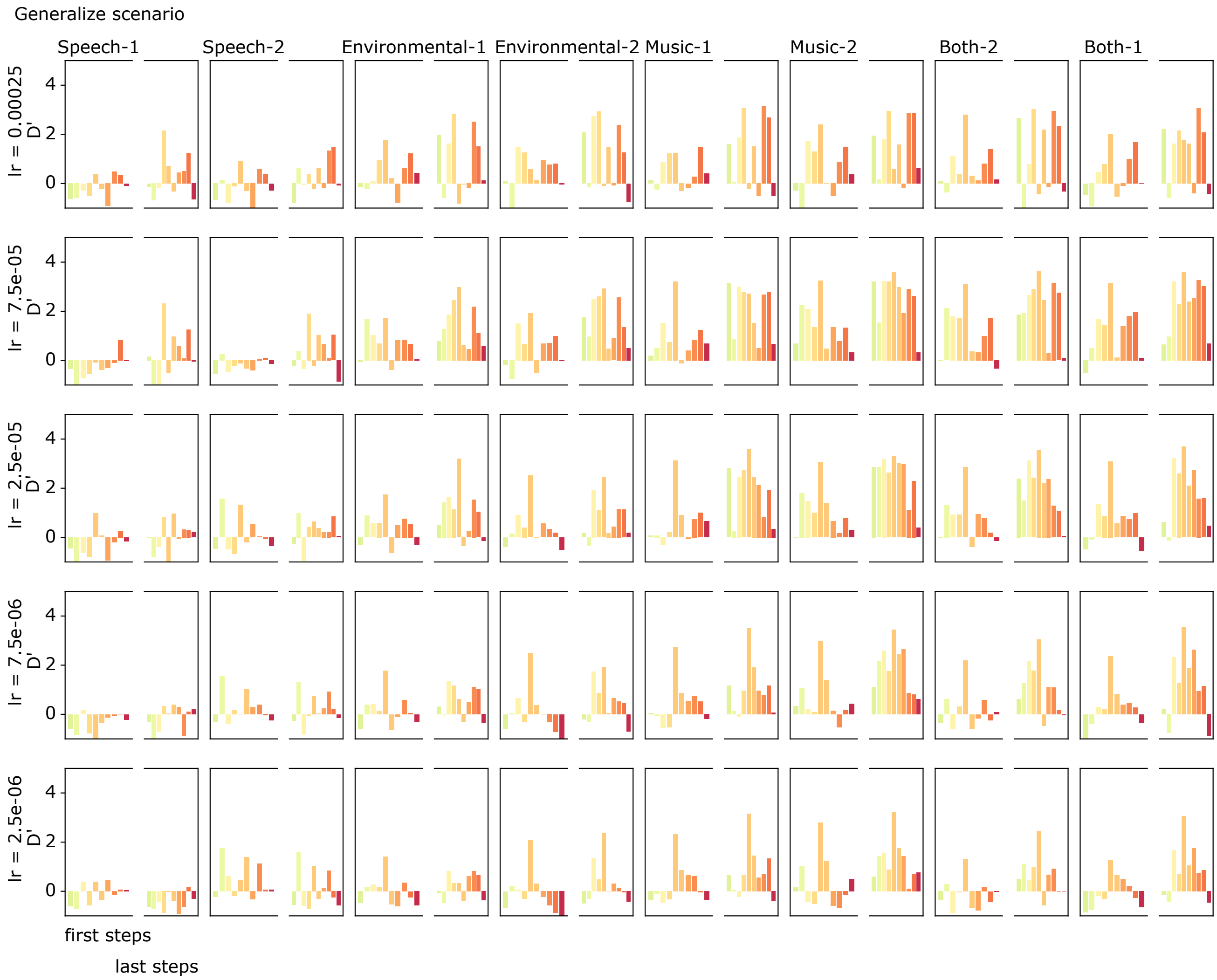

862

Supplementary Figure N. Test of the ability to generalize the algebraic patterns, with ISI = 250 ms. We measure the average model sensitivity across the early 5 to 50 steps of exposition (left), or the late 50 to 600 steps of exposition (right), for all models and 5 different learning rates (lr). The models are tested on the novel set of 10 sequences presented in Fig N in S1 Material, but in the case of the generalization scenario introduced in Fig E in S1 Material.

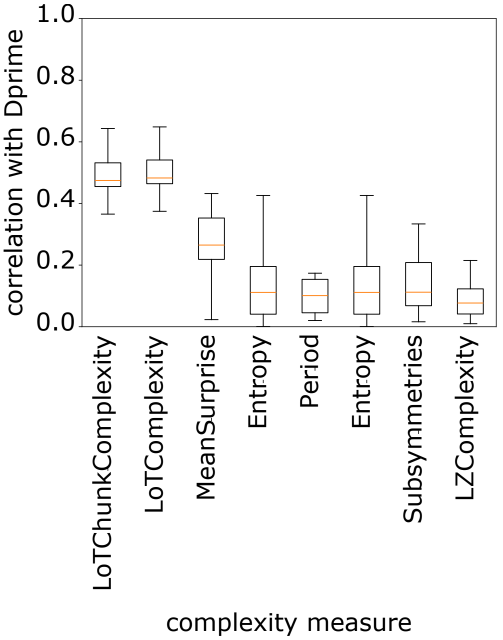

Supplementary Figure O. Correlation of different complexity metrics with the detection score D'. The model is tested with a few-shot protocol and is the same as in supplementary figure K. The model is tested on the algebraic pattern paradigms of (8). In this case, we save the model score every 50 steps of exposition and plot the average correlation across these steps.

Reference

1. Jackendoff R, Pinker S. The nature of the language faculty and its implications for evolution of language (reply to Fitch, Hauser, and Chomsky). Cognition. 2005;97(2):211-25. Place: Netherlands Publisher: Elsevier Science.
2. Berwick RC, Chomsky N. Why only us: language and evolution. First mit press paperback edition ed. Cambridge, Massachusetts London, England: The MIT Press; 2017.
3. Patel AD. Language, music, syntax and the brain. Nature Neuroscience. 2003 Jul;6(7):674-81. Number: 7 Publisher: Nature Publishing Group. Available from: https://www.nature.com/articles/nn1082.
4. Dehaene S, Meyniel F, Wacongne C, Wang L, Pallier C. The Neural Representation of Sequences: From Transition Probabilities to Algebraic Patterns and Linguistic Trees. Neuron. 2015 Oct;88(1):2-19. Publisher: Elsevier. Available from: https://www.cell.com/neuron/abstract/S0896-6273(15)00776-X.
5. Landemard A, Bimbard C, Demené C, Shamma S, Norman-Haignere S, Boubenec Y. Distinct higher-order representations of natural sounds in human and ferret auditory cortex. eLife. 2021 Nov;10:e65566. Publisher: eLife Sciences Publications, Ltd. Available from: https://doi.org/10.7554/eLife.65566.
6. Pinker S. How the mind works. 1st ed. Norton paperback. New York: Norton; 1999.
7. Marcus GF. The algebraic mind: integrating connectionism and cognitive science. Learning, development, and conceptual change. Cambridge, Mass London: MIT; 2001.
8. Al Roumi F, Planton S, Wang L, Dehaene S. Brain-imaging evidence for compression of binary sound sequences in human memory. eLife. 2023 Nov;12:e84376. Publisher: eLife Sciences Publications, Ltd. Available from: https://doi.org/10.7554/eLife.84376.
9. Sablé-Meyer M, Fagot J, Caparos S, van Kerkoerle T, Amalric M, Dehaene S. Sensitivity to geometric shape regularity in humans and baboons: A putative signature of human singularity. Proceedings of the National Academy of Sciences. 2021 Apr;118(16):e2023123118. Publisher: Proceedings of the National Academy of Sciences. Available from: https://www.pnas.org/doi/10.1073/pnas.2023123118.
10. Dehaene S, Al Roumi F, Lakretz Y, Planton S, Sablé-Meyer M. Symbols and mental programs: a hypothesis about human singularity. Trends in Cognitive Sciences. 2022 Sep;26(9):751-66. Available from: https://linkinghub.elsevier.com/retrieve/pii/S1364661322001413.
11. Kemp C, Tenenbaum JB. The discovery of structural form. Proceedings of the National Academy of Sciences. 2008 Aug;105(31):10687-92. Publisher: Proceedings of the National Academy of Sciences. Available from: https://www.pnas.org/doi/10.1073/pnas.0802631105.
12. Chomsky N. The Minimalist program. No. 28 in Current studies in linguistics. Cambridge, Mass: The MIT Press; 1995.
13. Bates E, Benigni L, Bretherton I, Camaioni L, Volterrra V. The emergence of symbols: cognition and communication in infancy. Language, thought, and culture. New York: Academic Press; 1979.
14. Saffran JR, Aslin RN, Newport EL. Statistical Learning by 8-Month-Old Infants. Science. 1996 Dec;274(5294):1926-8. Publisher: American Association for the Advancement of Science. Available from: https://www.science.org/doi/abs/10.1126/science.274.5294.1926.
15. Peña M, Bonatti LL, Nespor M, Mehler J. Signal-Driven Computations in Speech Processing. Science. 2002 Oct;298(5593):604-7. Publisher: American Association for the Advancement of Science. Available from: https://www.science.org/doi/10.1126/science.1072901.
16. Fiser J, Aslin RN. Statistical learning of new visual feature combinations by infants. Proceedings of the National Academy of Sciences. 2002 Nov;99(24):15822-6. Publisher: Proceedings of the

National Academy of Sciences. Available from: https://www.pnas.org/doi/abs/10.1073/pnas.232472899.

17. Schapiro AC, Rogers TT, Cordova NI, Turk-Browne NB, Botvinick MM. Neural representations of events arise from temporal community structure. Nature Neuroscience. 2013 Apr;16(4):486-92. Number: 4 Publisher: Nature Publishing Group. Available from: https://www.nature.com/articles/nn.3331.

18. Schapiro AC, Turk-Browne NB, Norman KA, Botvinick MM. Statistical learning of temporal community structure in the hippocampus. Hippocampus. 2016;26(1):3-8. _eprint: https://onlinelibrary.wiley.com/doi/pdf/10.1002/hipo.22523. Available from: https://onlinelibrary.wiley.com/doi/abs/10.1002/hipo.22523.

19. Benjamin L, Fló A, Al Roumi F, Dehaene-Lambertz G. Humans parsimoniously represent auditory sequences by pruning and completing the underlying network structure. eLife. 2023 May;12:e86430. Publisher: eLife Sciences Publications, Ltd. Available from: https://doi.org/10.7554/eLife.86430.

20. Näätänen R, Paavilainen P, Rinne T, Alho K. The mismatch negativity (MMN) in basic research of central auditory processing: a review. Clinical Neurophysiology: Official Journal of the International Federation of Clinical Neurophysiology. 2007 Dec;118(12):2544-90.

21. Bekinschtein TA, Dehaene S, Rohaut B, Tadel F, Cohen L, Naccache L. Neural signature of the conscious processing of auditory regularities. Proceedings of the National Academy of Sciences. 2009 Feb;106(5):1672-7. Publisher: Proceedings of the National Academy of Sciences. Available from: https://www.pnas.org/doi/10.1073/pnas.0809667106.

22. Wacongne C, Labyt E, Van Wassenhove V, Bekinschtein T, Naccache L, Dehaene S. Evidence for a hierarchy of predictions and prediction errors in human cortex. Proceedings of the National Academy of Sciences. 2011 Dec;108(51):20754-9. Available from: https://pnas.org/doi/full/10.1073/pnas.1117807108.

23. Barascud N, Pearce MT, Griffiths TD, Friston KJ, Chait M. Brain responses in humans reveal ideal observer-like sensitivity to complex acoustic patterns. Proceedings of the National Academy of Sciences. 2016 Feb;113(5):E616-25. Publisher: Proceedings of the National Academy of Sciences. Available from: https://www.pnas.org/doi/10.1073/pnas.1508523113.

24. Wacongne C, Changeux JP, Dehaene S. A neuronal model of predictive coding accounting for the mismatch negativity. The Journal of Neuroscience: The Official Journal of the Society for Neuroscience. 2012 Mar;32(11):3665-78.

25. Pearce MT. The construction and evaluation of statistical models of melodic structure in music perception and composition [doctoral]. City University London; 2005. Available from: https://openaccess.city.ac.uk/id/eprint/8459/.

26. Harrison PMC, Bianco R, Chait M, Pearce MT. PPM-Decay: A computational model of auditory prediction with memory decay. PLOS Computational Biology. 2020 Nov;16(11):e1008304. Publisher: Public Library of Science. Available from: https://journals.plos.org/ploscompbiol/article?id=10.1371/journal.pcbi.1008304.

27. Bianco R, Harrison PM, Hu M, Bolger C, Picken S, Pearce MT, et al. Long-term implicit memory for sequential auditory patterns in humans. eLife. 2020 May;9:e56073. Publisher: eLife Sciences Publications, Ltd. Available from: https://doi.org/10.7554/eLife.56073.

28. Lake B, Baroni M. Generalization without Systematicity: On the Compositional Skills of Sequence-to-Sequence Recurrent Networks. In: Proceedings of the 35th International Conference on Machine Learning. PMLR; 2018. p. 2873-82. ISSN: 2640-3498. Available from: https://proceedings.mlr.press/v80/lake18a.html.

29. Lake BM, Baroni M. Human-like systematic generalization through a meta-learning neural network. Nature. 2023 Nov;623(7985):115-21. Number: 7985 Publisher: Nature Publishing Group. Available from: https://www.nature.com/articles/s41586-023-06668-3.

30. Hupkes D, Dankers V, Mul M, Bruni E. Compositionality Decomposed: How do Neural Networks Generalise? Journal of Artificial Intelligence Research. 2020 Apr;67:757-95. Available from: https://www.jair.org/index.php/jair/article/view/11674.

31. Lakretz Y, Kruszewski G, Desbordes T, Hupkes D, Dehaene S, Baroni M. The emergence of number and syntax units in LSTM language models. arXiv; 2019. ArXiv:1903.07435 [cs]. Available from: http://arxiv.org/abs/1903.07435.

32. Linzen T, Dupoux E, Goldberg Y. Assessing the Ability of LSTMs to Learn Syntax-Sensitive Dependencies. Transactions of the Association for Computational Linguistics. 2016 Dec;4:521-35. Available from: https://doi.org/10.1162/tacl_a_00115.

33. McClelland JL, Botvinick MM, Noelle DC, Plaut DC, Rogers TT, Seidenberg MS, et al. Letting Structure Emerge: Connectionist and Dynamical Systems Approaches to Cognition. Trends in cognitive sciences. 2010 Aug;14(8):348-56. Available from: https://www.ncbi.nlm.nih.gov/pmc/articles/PMC3056446/.

34. Xie SM, Raghunathan A, Liang P, Ma T. An Explanation of In-context Learning as Implicit Bayesian Inference. arXiv; 2022. ArXiv:2111.02080 [cs]. Available from: http://arxiv.org/abs/2111.02080.

35. Chan S, Santoro A, Lampinen A, Wang J, Singh A, Richemond P, et al. Data Distributional Properties Drive Emergent In-Context Learning in Transformers. Advances in Neural Information Processing Systems. 2022 Dec;35:18878-91. Available from: https://proceedings.neurips.cc/paper_files/paper/2022/hash/77c6ccacfd9962e2307fc64680fc5ace-Abstract-Conference.html.

36. Brown T, Mann B, Ryder N, Subbiah M, Kaplan JD, Dhariwal P, et al. Language Models are Few-Shot Learners. In: Advances in Neural Information Processing Systems. vol. 33. Curran Associates, Inc.; 2020. p. 1877-901. Available from: https://papers.nips.cc/paper/2020/hash/1457c0d6bfcb4967418bfb8ac142f64a-Abstract.html.

37. Zhuang C, Xiang Z, Bai Y, Jia X, Turk-Browne N, Norman K, et al. How Well Do Unsupervised Learning Algorithms Model Human Real-time and Life-long Learning? Advances in Neural Information Processing Systems. 2022 Dec;35:22628-42. Available from: https://proceedings.neurips.cc/paper_files/paper/2022/hash/8dfc3a2720a4112243a285b98e0d4415-Abstract-Datasets_and_Benchmarks.html.

38. Zhuang C, Yan S, Nayebi A, Schrimpf M, Frank MC, DiCarlo JJ, et al. Unsupervised neural network models of the ventral visual stream. Proceedings of the National Academy of Sciences. 2021 Jan;118(3):e2014196118. Publisher: Proceedings of the National Academy of Sciences. Available from: https://www.pnas.org/doi/abs/10.1073/pnas.2014196118.

39. Millet J, Caucheteux C, Orhan P, Boubenec Y, Gramfort A, Dunbar E, et al. Toward a realistic model of speech processing in the brain with self-supervised learning. Advances in Neural Information Processing Systems. 2022 Dec;35:33428-43. Available from: https://proceedings.neurips.cc/paper_files/paper/2022/hash/d81ecfc8fb18e833a3fa0a35d92532b8-Abstract-Conference.html.

40. Testolin A, Stoianov I, Zorzi M. Letter perception emerges from unsupervised deep learning and recycling of natural image features. Nature Human Behaviour. 2017 Sep;1(9):657-64. Publisher: Nature Publishing Group. Available from: https://www.nature.com/articles/s41562-017-0186-2.

41. Baevski A, Zhou H, Mohamed A, Auli M. wav2vec 2.0: A Framework for Self-Supervised Learning of Speech Representations. arXiv; 2020. ArXiv:2006.11477 [cs, eess]. Available from: http://arxiv.org/abs/2006.11477.

42. Panayotov V, Chen G, Povey D, Khudanpur S. Librispeech: An ASR corpus based on public domain audio books. In: 2015 IEEE International Conference on Acoustics, Speech and Signal Processing (ICASSP); 2015. p. 5206-10. ISSN: 2379-190X. Available from: https://ieeexplore.ieee.org/document/7178964.

43. Defferrard M, Benzi K, Vandergheynst P, Bresson X. FMA: A Dataset For Music Analysis. arXiv; 2017. ArXiv:1612.01840 [cs]. Available from: http://arxiv.org/abs/1612.01840.

44. Gemmeke JF, Ellis DPW, Freedman D, Jansen A, Lawrence W, Moore RC, et al. Audio Set: An ontology and human-labeled dataset for audio events. In: 2017 IEEE International Conference on Acoustics, Speech and Signal Processing (ICASSP); 2017. p. 776-80. ISSN: 2379-190X. Available from: https://ieeexplore.ieee.org/document/7952261.

45. King JR, Gramfort A, Schurger A, Naccache L, Dehaene S. Two Distinct Dynamic Modes Subtend the Detection of Unexpected Sounds. PLoS ONE. 2014 Jan;9(1):e85791. Available from: https://dx.plos.org/10.1371/journal.pone.0085791.

46. King JR, Faugeras F, Gramfort A, Schurger A, El Karoui I, Sitt JD, et al. Single-trial decoding of auditory novelty responses facilitates the detection of residual consciousness. NeuroImage. 2013 Dec;83:726-38. Available from: https://linkinghub.elsevier.com/retrieve/pii/S1053811913007684.

47. Basirat A, Dehaene S, Dehaene-Lambertz G. A hierarchy of cortical responses to sequence violations in three-month-old infants. Cognition. 2014 Aug;132(2):137-50.

48. El Karoui I, King JR, Sitt J, Meyniel F, Van Gaal S, Hasboun D, et al. Event-Related Potential, Time-frequency, and Functional Connectivity Facets of Local and Global Auditory Novelty Processing: An Intracranial Study in Humans. Cerebral Cortex. 2015 Nov;25(11):4203-12. Available from: https://academic.oup.com/cercor/article-lookup/doi/10.1093/cercor/bhu143.

49. Todorovic A, Lange FPd. Repetition Suppression and Expectation Suppression Are Dissociable in Time in Early Auditory Evoked Fields. Journal of Neuroscience. 2012 Sep;32(39):13389-95. Publisher: Society for Neuroscience Section: Articles. Available from: https://www.jneurosci.org/content/32/39/13389.

50. Uhrig L, Dehaene S, Jarraya B. A Hierarchy of Responses to Auditory Regularities in the Macaque Brain. Journal of Neuroscience. 2014 Jan;34(4):1127-32. Publisher: Society for Neuroscience Section: Brief Communications. Available from: https://www.jneurosci.org/content/34/4/1127.

51. Jiang Y, Komatsu M, Chen Y, Xie R, Zhang K, Xia Y, et al. Constructing the hierarchy of predictive auditory sequences in the marmoset brain. eLife. 2022 Feb;11:e74653. Publisher: eLife Sciences Publications, Ltd. Available from: https://doi.org/10.7554/eLife.74653.

52. Oota SR, Çelik E, Deniz F, Toneva M. Speech language models lack important brain-relevant semantics. arXiv; 2023. ArXiv:2311.04664 [cs, eess, q-bio]. Available from: http://arxiv.org/abs/2311.04664.

53. Fedorenko E, Behr MK, Kanwisher N. Functional specificity for high-level linguistic processing in the human brain. Proceedings of the National Academy of Sciences. 2011 Sep;108(39):16428-33. Publisher: Proceedings of the National Academy of Sciences. Available from: https://www.pnas.org/doi/abs/10.1073/pnas.1112937108.

54. Radford A, Kim JW, Xu T, Brockman G, McLeavey C, Sutskever I. Robust Speech Recognition via Large-Scale Weak Supervision. arXiv; 2022. ArXiv:2212.04356 [cs, eess]. Available from: http://arxiv.org/abs/2212.04356.

55. Défossez A, Copet J, Synnaeve G, Adi Y. High Fidelity Neural Audio Compression. arXiv; 2022. ArXiv:2210.13438 [cs, eess, stat]. Available from: http://arxiv.org/abs/2210.13438.

56. Hu M, Bianco R, Hidalgo AR, Chait M. Concurrent Encoding of Precision and Prediction Error in Unfolding Auditory Patterns: Insights from MEG. bioRxiv; 2023. Pages: 2023.10.06.561171 Section: New Results. Available from: https://www.biorxiv.org/content/10.1101/2023.10.06.561171v1.

57. Agus TR, Pressnitzer D. The detection of repetitions in noise before and after perceptual learning. The Journal of the Acoustical Society of America. 2013 Jul;134(1):464-73. Available from: https://doi.org/10.1121/1.4807641.

58. Agus TR, Thorpe SJ, Pressnitzer D. Rapid Formation of Robust Auditory Memories: Insights from Noise. Neuron. 2010 May;66(4):610-8. Available from: https://www.sciencedirect.com/science/article/pii/S0896627310002850.

59. Kang H, Agus TR, Pressnitzer D. Auditory memory for random time patterns. The Journal of the Acoustical Society of America. 2017 Oct;142(4):2219.

60. Marr D. A theory for cerebral neocortex. Proceedings of the Royal Society of London Series B, Biological Sciences. 1970 Nov;176(1043):161-234.

61. G B. Two-stage model of memory trace formation: a role for "noisy" brain states. Neuroscience. 1989;31(3). Publisher: Neuroscience. Available from: https://pubmed.ncbi.nlm.nih.gov/2687720/.

62. Battaglia FP, Benchenane K, Sirota A, Pennartz CMA, Wiener SI. The hippocampus: hub of brain network communication for memory. Trends in Cognitive Sciences. 2011 Jul;15(7):310-8. Publisher: Elsevier. Available from: https://www.cell.com/trends/cognitive-sciences/abstract/S1364-6613(11)00089-1.

63. Kumar S, Bonnici HM, Teki S, Agus TR, Pressnitzer D, Maguire EA, et al. Representations of specific acoustic patterns in the auditory cortex and hippocampus. Proceedings of the Royal Society B: Biological Sciences. 2014 Sep;281(1791):20141000. Publisher: Royal Society. Available from: https://royalsocietypublishing.org/doi/10.1098/rspb.2014.1000.

64. Wang L, Uhrig L, Jarraya B, Dehaene S. Representation of Numerical and Sequential Patterns in Macaque and Human Brains. Current Biology. 2015 Aug;25(15):1966-74. Available from: https://linkinghub.elsevier.com/retrieve/pii/S0960982215007290.

65. Jamali S, Bagur S, Brémont E, Kerkoerle TV, Dehaene S, Bathellier B. Parallel mechanisms signal a hierarchy of sequence structure violations in the auditory cortex. bioRxiv; 2024. Pages: 2024.08.21.609026 Section: New Results. Available from: https://www.biorxiv.org/content/10.1101/2024.08.21.609026v1.

66. Poole KC. How does the brain extract acoustic patterns? A behavioural and neural study [Doctoral]. UCL (University College London); 2023. Publication Title: Doctoral thesis, UCL (University College London). Available from: https://discovery.ucl.ac.uk/id/eprint/10173385/.

67. Gilkerson J, Richards JA, Warren SF, Montgomery JK, Greenwood CR, Kimbrough Oller D, et al. Mapping the Early Language Environment Using All-Day Recordings and Automated Analysis. American Journal of Speech-Language Pathology. 2017 May;26(2):248-65. Available from: http://pubs.asha.org/doi/10.1044/2016_AJSLP-15-0169.

68. Hewitt J, Manning CD. A Structural Probe for Finding Syntax in Word Representations. In: Proceedings of the 2019 Conference of the North. Minneapolis, Minnesota: Association for Computational Linguistics; 2019. p. 4129-38. Available from: http://aclweb.org/anthology/N19-1419.

69. Li Y, Anumanchipalli GK, Mohamed A, Chen P, Carney LH, Lu J, et al. Dissecting neural computations in the human auditory pathway using deep neural networks for speech. Nature Neuroscience.

2023 Dec;26(12):2213-25. Available from: https://www.nature.com/articles/s41593-023-01468-4.

70. Vaidya AR, Jain S, Huth AG. Self-supervised models of audio effectively explain human cortical responses to speech. arXiv; 2022. Version Number: 1. Available from: https://arxiv.org/abs/2205.14252.

71. Francl A, McDermott JH. Deep neural network models of sound localization reveal how perception is adapted to real-world environments. Nature Human Behaviour. 2022 Jan;6(1):111-33. Publisher: Nature Publishing Group. Available from: https://www.nature.com/articles/s41562-021-01244-z.

72. Tuckute G, Feather J, Boebinger D, McDermott JH. Many but not all deep neural network audio models capture brain responses and exhibit correspondence between model stages and brain regions. PLOS Biology. 2023 Dec;21(12):e3002366. Available from: https://dx.plos.org/10.1371/journal.pbio.3002366.

73. Kim G, Kim DK, Jeong H. Spontaneous emergence of rudimentary music detectors in deep neural networks. Nature Communications. 2024 Jan;15(1):148. Publisher: Nature Publishing Group. Available from: https://www.nature.com/articles/s41467-023-44516-0.

74. LeCun Y, Boser B, Denker J, Henderson D, Howard R, Hubbard W, et al. Handwritten Digit Recognition with a Back-Propagation Network. In: Advances in Neural Information Processing Systems. vol. 2. Morgan-Kaufmann; 1989. Available from: https://proceedings.neurips.cc/paper/1989/hash/53c3bce66e43be4f209556518c2fcb54-Abstract.html.

75. Wolf T, Debut L, Sanh V, Chaumond J, Delangue C, Moi A, et al. Transformers: State-of-the-Art Natural Language Processing. In: Proceedings of the 2020 Conference on Empirical Methods in Natural Language Processing: System Demonstrations. Online: Association for Computational Linguistics; 2020. p. 38-45. Available from: https://aclanthology.org/2020.emnlp-demos.6.

76. Wesker T, Meyer B, Wagener K, Anemüller J, Mertins A, Kollmeier B. Oldenburg logatome speech corpus (OLLO) for speech recognition experiments with humans and machines. In: Interspeech 2005. ISCA; 2005. p. 1273-6. Available from: https://www.isca-speech.org/archive/interspeech_2005/wesker05_interspeech.html.

77. Tzanetakis G, Cook P. Musical genre classification of audio signals. IEEE Transactions on Speech and Audio Processing. 2002 Jul;10(5):293-302. Available from: https://ieeexplore.ieee.org/document/1021072/.

78. Sturm BL. The GTZAN dataset: Its contents, its faults, their effects on evaluation, and its future use. Journal of New Music Research. 2014 Apr;43(2):147-72. ArXiv:1306.1461 [cs]. Available from: http://arxiv.org/abs/1306.1461.

79. Piczak KJ. ESC: Dataset for Environmental Sound Classification. In: Proceedings of the 23rd ACM international conference on Multimedia. MM '15. New York, NY, USA: Association for Computing Machinery; 2015. p. 1015-8. Available from: https://doi.org/10.1145/2733373.2806390.

80. McFee B, McVicar M, Faronbi D, Roman I, Gover M, Balke S, et al.. librosa/librosa: 0.10.1. Zenodo; 2023. Available from: https://zenodo.org/record/8252662.

81. Luccioni AS, Viguier S, Ligozat AL. Estimating the Carbon Footprint of BLOOM, a 176B Parameter Language Model. arXiv; 2022. ArXiv:2211.02001 [cs]. Available from: http://arxiv.org/abs/2211.02001.

82. Planton S, Kerkoerle Tv, Abbih L, Maheu M, Meyniel F, Sigman M, et al. A theory of memory for binary sequences: Evidence for a mental compression algorithm in humans. PLOS Computational Biology. 2021 Jan;17(1):e1008598. Publisher: Public Library of Science. Available from: https://journals.plos.org/ploscompbiol/article?id=10.1371/journal.pcbi.1008598.

83. Raffel C. Learning-Based Methods for Comparing Sequences, with Applications to Audio-to-MIDI Alignment and Matching. 2016. Publisher: Columbia University. Available from: https://academiccommons.columbia.edu/doi/10.7916/D8N58MHV.

84. Lempel A, Ziv J. On the Complexity of Finite Sequences. IEEE Transactions on Information Theory. 1976 Jan;22(1):75-81. Available from: http://ieeexplore.ieee.org/document/1055501/.

85. Zhang Y, Hao J, Zhou C, Chang K. Normalized Lempel-Ziv complexity and its application in bio-sequence analysis. Journal of Mathematical Chemistry. 2009 Nov;46(4):1203-12. Available from: http://link.springer.com/10.1007/s10910-008-9512-2.
