# Supplementary material for: The detection of algebraic auditory structures emerges with self-supervised learning
Source: PLoS Comput Biol. 2025 Sep 5;21(9):e1013271. doi: 10.1371/journal.pcbi.1013271 (PMC12431648; doi:10.1371/journal.pcbi.1013271)
Supplement: Attachment — Submitted filename: PlosBioRebutal.pdf [file pcbi.1013271.s002.pdf]

Summary:

We thank our three reviewers for their constructive comments.

Overall, the study was deemed “highly engaging” (R1), “interesting”(R3), “rigorous in methodology” (R1), and “appealing to a broad audience” (R2), using a “novel approach”(R1).

Nevertheless, the reviewers rightfully raised five main concerns, which we now address with novel experiments.

First, all reviewers questioned if the advantage of the music models could be explained by the use of tones in most experiments. To address this issue, we now extend our experiments to a 3x3x4 design, where all **3** models (music, speech, environmental) are tested with all **3** types of sounds (tones, artificial syllables, and, novel, excerpt of environmental sounds) on all **4** protocols. The results of these experiments confirm our original conclusions.

Second, R1 stressed an issue in Fig4, with the decoding score on the repeat sentence smaller than theoretically expected. We went back to this analysis and discovered that the decoder overfited due to poor balancing of high versus low tones. Changing the evaluation to a balanced dataset prevented the decoder from overfitting. This led to clearer decoding curves without changing the conclusion of this decoding analysis.

Third, R2 questioned if stressed syllables could help structure detection in the speech models. We perform this experiment and show that this does not change the model behavior, strengthening our initial conclusion.

Fourth, R3 questioned the use of the Audioset, as it has several drawbacks, including poor sound quality and annotations. We clarified the exact filter used to remove audio files that contained speech and music. Additionally, we performed a manual verification of the quality of this filtering. Although there remain some music files in the training dataset, we measure that they are in small numbers compared to temporally structured environmental sounds. We stress this issue in the manuscript.

Fifth, R3 questioned if our results were dependent on the optimization parameters and architecture used to train the models. To test the effect of architectural differences, we trained speech and music models of different sizes and confirmed that these variations had little effect on the model behavior.

We would like to thank our reviewers for their thorough review which helps improve both the clarity and the conclusions of our study.

Reviewer #1:

Reviewer's Responses to Questions

In this work the authors set out to study whether representations of algebraic auditory structures can emerge in a model trained in a purely self-supervised fashion without enforcing specific rules and constraints to make it possible. In the bigger picture, the answer to this question aims to address the bigger debate between "radicalist" and "empiricist" views on how humans learn to detect abstract structures in sound and other sensory modalities. The authors take a novel approach to the problem by using zero-shot contrastive loss of individually masked units of sound to measure model surprisal to stimuli, and then using that surprisal to emulate psychoacoustic experimental paradigms to measure the abstraction of auditory structures as studied in humans. The results of the study make a strong argument for the empiricist view.

Overall, I found the paper highly engaging in terms of novelty of approach and results, and rigorous in methodology. I did not come across any major issues, but I do have a few minor comments that I hope will serve to improve the manuscript even further.

1.0

Minor comments:

1. One of the most interesting parts of the results for me was the finding that models trained on speech alone did not learn to extract abstract structures. While the authors have dedicated a section to this in the results and also address it in the Discussion and supplemental materials, I think there is space for more exploration here. There are a few examples below. I do not expect them to be addressed with new experiments, but I think some more general discussion on this point is in order.
a. The authors name one possible reason for the finding which is that the speech dataset used for training is not as "repetitive" as the music dataset, which they also show quantitatively. Does this suggest if a more repetitive speech dataset was used for training, like a larger version of the TIMIT dataset, it would show a higher degree of abstraction? What about songs?

Author response

We agree with the reviewer that our results predict that training on a repetitive speech dataset would likely favor the emergence of auditory structure, yet this remains an expensive experiment to perform, and we believe more progress would be achieved by better understanding the code underlying structure detection.
Although songs are sometimes repetitive, their effect would be difficult to disambiguate from the melody that accompanies them. Furthermore, the main interest in differentiating speech and music is to discover which type of structure (musical versus linguistic) in the training dataset favors the emergence of an online structure detection algorithm. With songs, this would be difficult to precisely assess.

1.1

b. The authors show that all four models trained perform reasonably well on some high-level classification tasks involving speech, natural sounds, and music (Suppl Fig 8), but these are perhaps too high-level. For example an automatic speech recognition model will learn to ignore most sources of non-speech sounds as they are noise with respect to the task of speech recognition. Similarly, the Wav2Vec2 trained on speech might be suppressing/normalizing the non-speech sounds in the stimulus, which would include most of the experiment data which consist of tones. One way to test that this is not the case is to measure the mask reconstruction accuracy of the speech model on a few very simple patterns of alternating tones (ABABAB...) since that is the stimulus for 3/4 tests. Something like this would show if the speech model is capable of sufficiently representing the building blocks of the test stimuli.

Author response

We acknowledge that it could have been possible that the speech model ignores specific types of sounds. Nevertheless, we ran during our exploration several tests to confirm this was not the case. At a lower level we observed that, at the output of the convolution layers, the activities of models trained on speech, music or environmental sounds were very similar by performing a correlation of the response-similarity matrix of their activity in response to every sound element. For the ABAB patterns (without masking the stimuli) this was evident by a checkerboard-like RSA matrix, demonstrating that all models sufficiently contrasted the two tones in the sound sequence.

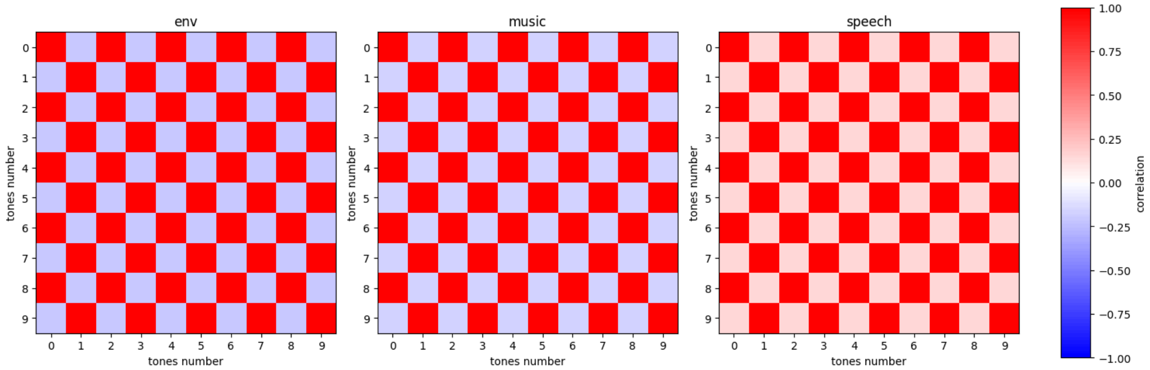

Rebuttal figure 1: Representation similarity matrices of the activity of the last convolution layers, in response to sequences of alternating tones. Note the checkerboard pattern revealing that tones are well separated in the model activity space.

1.2

c. Connected to point (b) above, the tests are made up of sequences of tones that are mostly found in music. Could this be the reason why musical exposure shows the biggest promise? What if the stimulus units in all cases were syllables or pieces of stationary environmental sounds?

Author response The reviewer is right that the nature of the test stimulus could act as a confound in the subsequent analysis. To control for this, we extend our tests to a 3x3x4 design, where all **3** models (music, speech, environmental) are tested with all **3** types of sounds (novel excerpts of environmental sounds, artificial syllables, and tones) on all **4** protocols.

To summarize our results, we report the surprisal to rand metric (similar to Fig 2, C,F,I,L) for each of these conditions. Speech models systematically failed to perform structure detection in all 3x4 conditions, in stark contrast with the music and environmental sounds model. This experiment addresses the same concerns of R1, R2, and R3.

test stimulus	model	AlRoumi2023	Barascud2016	Saffran1996	Bekinschtein2009
natural sound	environmental pretraining	2.50	5.45	4.43	1.58
	music pretraining	7.41	5.88	3.83	6.58
	speech pretraining	**0.16**	**0.48**	**0.02**	**-0.02**
speech	environmental pretraining	0.20	1.96	1.92	0.10
	music pretraining	0.23	2.39	2.26	0.97
	speech pretraining	**-0.04**	**0.17**	**-0.06**	**-0.10**
tones	environmental pretraining	0.76	1.45	1.69	1.68
	music pretraining	4.68	4.50	2.90	4.17
	speech pretraining	**-0.02**	**0.27**	**-0.26**	**-0.19**

Table 2: Cross dataset evaluation: We evaluate each model on the 4 types of sequences changing the sequence elements to environmental sound, syllables, or tones. In all situations, speech models are never able to detect the auditory structure. This shows that the difference between the models is not explained by a difference in the type of test elements.

1.3

d. The results for the models trained individually on the three types of data are compared, but I would also suggest comparing results between the model trained on music only vs the one trained on a balanced dataset.

Author response:

The model trained on the balanced dataset had a behavior that was intermediate to the model trained on music and the model trained on environmental sounds.

1.4

2. On lines 201-202 of the manuscript, I did not follow how the authors came to the conclusion that "3 months is therefore an upper limit on the amount of data needed for this detection". Did this follow from the finding that after 3 (audio-)months of pre-training the model it was able to detect global deviant - local standard? Does this not depend on data variety, loss function, and model architecture/size? Or did it follow from the study that showed children at 3 months old can learn such patterns? Either way, I think the sentence could use further clarification.

Author response:
We thank the reviewer for asking to clarify this sentence. Indeed, this conclusion is dependent on the data variety, loss function and model architecture/size.

To clarify, we removed this problematic sentence and changed the paragraph to be the following:

Although the synthetic environment differs considerably from the continuous sound stream heard by the children, it remains that the model can learn from at least 3 months of data, which is reasonable at least in order of magnitude.

1.5

3. There is a surprising result in Figure 4 that is worth mentioning. And that is the case of the simplest algebraic pattern – repeat. To my understanding the decoder here is a binary classifier, which makes the random baseline accuracy 50%, correct? So when the decoding accuracy is ~5% for the first transformer layer, that means if you flip the prediction it would be correct 95% of the time. Is this a bug, is the dataset that this decoding is being tested on unbalanced, or something else? Because as is, it looks like the simplest algebraic pattern emerges deepest in the model, if we define emergence as crossing threshold of X% accuracy where X < 90.

Author response:
We thank the reviewer for having correctly pointing out this discrepancy. It indeed comes from an unbalance we had overlooked. In the training set, we used 10 sequences and their complementary, which involved a total of 20 different frequency tones.
The problem came from the fact that we used for the A tones only high frequency tones instead of alternating between high and low frequency effect. This created a strong overfitting cues which was catastrophic for the "repeat" sequence, notably because these sequences confounded the frequency height with the deviant tones: deviant was always the B tones, which was always a "low" tone. This effect was less severe in other sequences, as the deviant tones could be both the A and B tones.
To resolve this issue, we changed our training and test splits, alternating between high and low frequency for the A tones, while still using the exact complementary version of each train sequence as a test sequence.

We clarify the result section accordingly:

“For that purpose we generated for each sequence type 10 trials and their complementary.
For each train trial, the A tones was taken from 5 possible high tones and 5 other possible low tones.
For each test sequence, the A tones was taken from 5 other possible high tones and 5 other possible low tones. Finally, we added in the test trials each complementary trial from the train trials, and from the train trials each complementary trial of the test trials. This prevented overfitting of the decoder to tone frequencies”

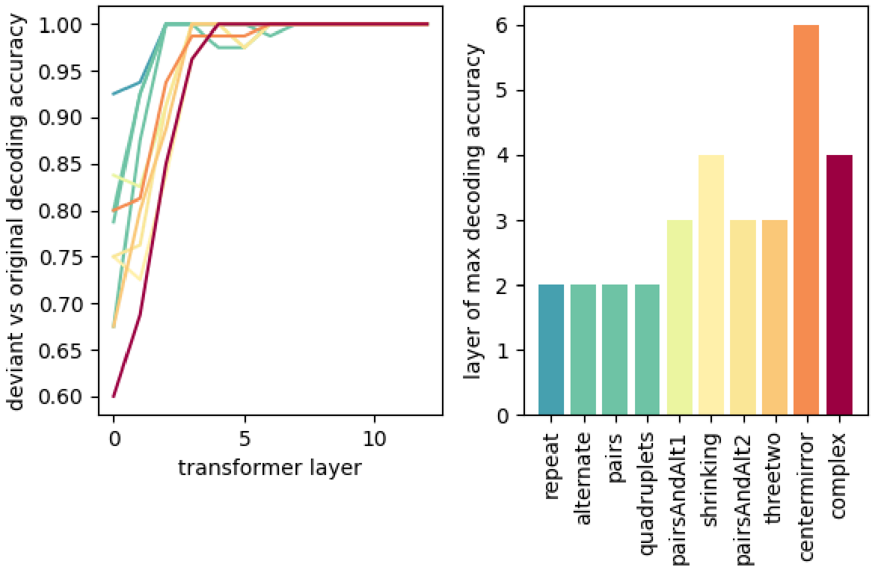

Figure 4, Corrected: Decoding regular vs non-regular for each model layer. Left: decoding accuracy of deviant versus original tones. The classifier is learned over all types of sequences and evaluation is separated across different types of sequences. The dataset are composed of 1000 sequences, with the 500 test sequences being the exact complementary to the training sequences (inversion of the role of each tone). Deviant tones are detected in earlier layers for simpler sequences. Right: earliest layer at which the decoding accuracy is maximal, for each type of sequence.

4. While the scientific content of the article was quite engaging to read, I think the language could have been improved in a few places. Specifically cases of grammar mistakes, typos, and sentences that were too long. These made the paper difficult to follow at times.

Author response:

We fixed some typos and grammatical mistakes.

Reviewer #2:

This paper investigates whether Wav2Vec2, a self-supervised deep learning model, can detect complex auditory structures similar to human cognitive abilities when trained on natural speech, music, or environmental sounds. The findings demonstrate that the models progressively acquired the ability to detect increasingly complex auditory structures with continued training. Notably, this ability did not emerge when the model was trained solely on natural speech and developed more rapidly with music pretraining compared to environmental sounds. The paper situates itself within the "rationalists vs. empiricists" debate and appeals to a broad audience in cognitive science. I'm generally positive for endorsing this paper for publication, but the following comments should be addressed before publication.

Major:

1.1

1. The syllable stimuli used for testing word segmentation were generated with a synthesizer, with pitch removed to mirror the original setting of Saffran et al. (1996). However, subsequent work by (Yang, 2004) has shown that stress patterns play a more significant role than transitional probabilities between syllables in real word segmentation. This could explain the model's failure to perform chunking when trained solely on speech data. It would be interesting to see how the model performs with three-syllable sequences featuring primary stress on the first syllable.

Author response:
We agree with the reviewer that the importance of stress on syllables for word segmentation, as interestingly underlined in Yang, 2004. One could hypothesize that a speech recognition system could perform partial word segmentation without any repetition detection algorithm. To test this hypothesis, we generated three-syllable sequences featuring a primary stress pattern. By definition, a stressed syllable has a longer, louder, and higher sound than the other syllables in the word. We chose to manipulate stress by lengthening the duration of the first syllable. More precisely we halved the speed used to generate this syllable in the Mbrola synthesizer. Despite these changes, the speech model remained unable to detect the repetition, stressing once more that speech models do not perform an analysis of the statistical structure of their input stimuli.

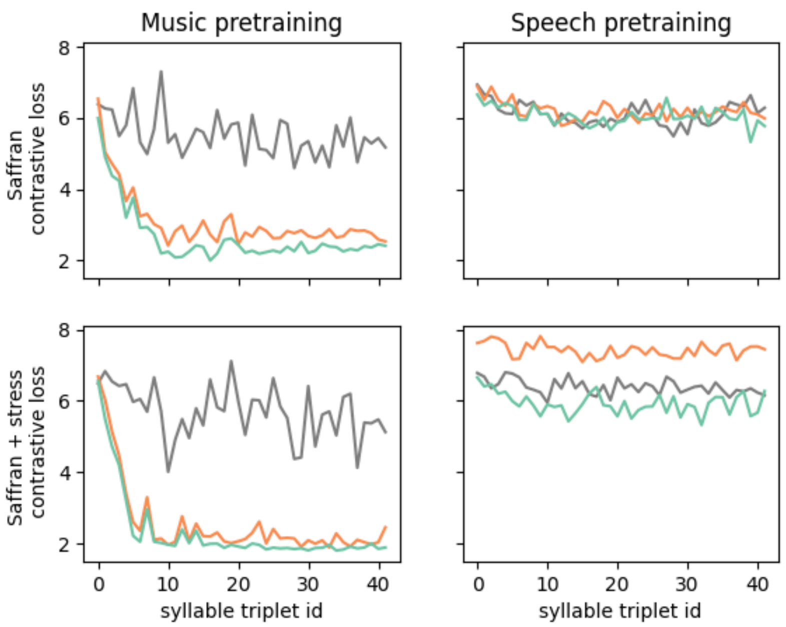

Rebuttal figure 2: Responses of a speech (right) and music-exposed (left) models to 10 repetitions of the classical Saffran paradigms (top) and of a modified Saffran paradigm (bottom), where the first syllable of each word is stressed.

1.2

2. The finding that music pretraining led to better structure detection compared to speech and environmental sounds is intriguing but lacks a sufficient explanation. Is there any metric available to quantify the similarity between the training data and the test stimuli? Could it be that the music dataset is, in fact, more similar to the synthesized test stimuli?

Author response:

The reviewer is right that this is a surprising finding, and our explanation propose that it is the amount of regularity that matters. To better control that the acoustic similarity played no role in this finding, we extend our tests to a 3x3x4 design , where all **3** models (music, speech, environmental) are tested with all **3** types of sounds (tones, artificial syllables, and, novel, excerpt of environmental sounds) on all **4** protocols. To summarize our results, we report the surprisal to rand metric (similar to Fig 2, C,F,I,L), for each of these conditions. Speech models systematically failed to perform structure detection in all 3x4 conditions, in stark contrast with the music and environmental sounds model. This experiment addresses the same concerns of R1, R2 and R3.

test stimulus	model	AlRoumi2023	Barascud2016	Saffran1996	Bekinschtein2009
natural sound	environmental pretraining	2.50	5.45	4.43	1.58
	music pretraining	7.41	5.88	3.83	6.58
	speech pretraining	**0.16**	**0.48**	**0.02**	**-0.02**
speech	environmental pretraining	0.20	1.96	1.92	0.10

	music pretraining	0.23	2.39	2.26	0.97
	speech pretraining	**-0.04**	**0.17**	**-0.06**	**-0.10**
tones	environmental pretraining	0.76	1.45	1.69	1.68
	music pretraining	4.68	4.50	2.90	4.17
	speech pretraining	**-0.02**	**0.27**	**-0.26**	**-0.19**

Table 2: Cross dataset evaluation: We evaluate each model on the 4 types of sequences changing the sequence elements to environmental sound, syllables, or tones. In all situations, speech models are never able to detect the auditory structure. This shows that the difference between the models is not explained by a difference in the type of test elements.

1.3

3. I understand that it is interesting to demonstrate how models trained on natural sounds can generalize to artificial structure detection. However, would it also be worthwhile to include results on the model's performance in detecting natural structures, such as syntactic constructions?

Author response:

We agree with the reviewer that the study of other structures, including linguistic ones, is a promising avenue. This was out-of-scope for this paper, which focused on artificial structures proposed in paradigms from the literature.

Minor:

2.1

1. The labels on all line plots in Figures 2 and 3 indicate "surprise" or "surprisal," while the captions describe the metric as the "difference between the mean contrastive loss." This seems to deviate from the canonical definition of "surprisal," typically defined as the negative log of a probability distribution. Additionally, the Methods section mentions the LOT complexity metric. Could you clarify where this metric was applied in the analyses?

Author response:

The reviewer is correct that the use of surprise differs from its typical definition. Nevertheless, this contrastive loss parallels the negative log of the softmax probability distribution used in other architectures. In this manner, we used this denomination to make the parallel easier and facilitate the reading.
The LOT complexity metric is used to compute the complexity of a binary sequence of tones, notably for the sequences from Al Roumi et al. as well as the novel set of sequences we test.

2. The authors deliberately exclude LLMs for their use of "unreasonably large and symbolic datasets." However, it might be valuable to include the performance of these LLMs on the testing data for comparison.

Yang, C. D. (2004). Universal Grammar, statistics or both? Trends in Cognitive Sciences, 8(10), 451–456.

Signed: Jixing Li

Author response:

We also decided to leave LLMs out of the work for the sake of clarity. The difference in training data might enable the LLM to develop a sequence processing algorithm that reflects symbolic language processing rather than the initial speech processing. This could confuse the reader on the relevance of speech exposition vs symbolic language for automatic auditory structure processing.

Furthermore, the processing of these auditory structures are known to involve different brain regions than the language network (AlRoumi et al 2023). The origin of their emergence is therefore unlikely to rely on purely linguistic processing. LLMs in turn perform a mix of linguistic and symbolic processing, and they would have not been useful in theorizing which source of data contributes in sequence processing.

Reviewer #3:

The paper is an interesting set of computational experiments studying how differences in self-supervised training of DNNs can lead to different emergent properties in detecting structure of the input. The authors relate these properties to well-studied phenomena found in humans that emerge over development. Overall, I found the paper interesting, but there are a few major concerns regarding possible confounds of the experiments that I think should be addressed prior to publication.

Major Concerns:

1.1

1) I am concerned that the reported results with environmental sound training is contaminated by the presence of music in the training dataset. The authors state that their model is trained on “environmental sounds” by excluding music and speech sounds from AudioSet using the taxonomy, however the AudioSet labels are sloppy and if a label is not present on a clip that does not guarantee that the category is *not* present in the clip (quality ratings for the labels are for positive labels). In fact, there are a large number of AudioSet clips containing music but not labeled as so. This has been pointed out for other similar studies, for instance: https://www.nature.com/articles/s41467-023-44516-0 where they report that in the *balanced* dataset about 4.5% of the clips have music (and I believe the listed clips are on their GitHub). I suspect this is even higher for the unbalanced dataset. Did the authors ensure that their training set has NO music? If not, to make the claim about the differences in training environments, I believe a new model must be trained on data known to contain no music (yes, I acknowledge this might be quite time-consuming, sorry). It unfortunately could be the case that it has some music but less which is driving the similarity to the musical training. Further, I could not find the list of categories that were excluded from AudioSet for training. This *must* be reported for reproducibility (for instance, were all of the musical instruments excluded? What about things like telephone ringtones?)

Author Response

The reviewer is right to be concerned about the quality of the AudioSet dataset. We quickly realized in the projects that sounds were poorly recorded and of low quality. At the time of datasets selection and cleaning (2021), we decided to filter purely based on the taxonomy proposed by the datasets. For that purpose, we used the ontology and filtered out of the dataset any sounds using the following labels:

Speech related labels:

```
['A capella' 'Babbling' 'Baby cry, infant cry' 'Baby laughter'
 'Battle cry' 'Birthday music' 'Bleat' 'Boing' 'Booing' 'Cacophony'
 'Chatter' 'Cheering' 'Child singing' 'Child speech, kid speaking'
 'Children playing' 'Children shouting' 'Choir' 'Chorus effect'
 'Christian music' 'Christmas music' 'Conversation' 'Crowd'
 'Crying, sobbing' 'Female singing' 'Female speech, woman speaking'
 'Hubbub, speech noise, speech babble' 'Human voice' 'Laughter'
 'Male singing' 'Male speech, man speaking' 'Rapping'
 'Ice cream truck, ice cream van' 'Inside, large room or hall'
 'Inside, public space' 'Inside, small room' 'Narration, monologue' 'Song'
```

```
 'Speech' 'Speech synthesizer' 'Telephone' 'Television' 'Yell' 'Yodeling'
 'Whispering' 'Whimper' 'Whoop']
```

Music related labels:

```
['A capella' 'Accordion' 'Acoustic guitar' 'Afrobeat' 'Alto saxophone'
 'Ambient music' 'Angry music' 'Background music' 'Bass (instrument role)'
 'Bass (frequency range)' 'Bass drum' 'Bass guitar' 'Bassline' 'Bassoon'
 'Beat' 'Beatboxing' 'Birthday music' 'Bugle' 'Blues'
 'Bowed string instrument' 'Brass instrument' 'Carnatic music' 'Cacophony'
 'Chime' 'Choir' 'Chord' 'Chorus effect' 'Christian music'
 'Christmas music' 'Chuckle, chortle' 'Clarinet' 'Classical music' 'Cello'
 'Cellphone buzz, vibrating alert' 'Chant' 'Cheering' 'Child singing'
 'Church bell' 'Clavinet' 'Compact disc' 'Country' 'Cornet' 'Crash cymbal'
 'Cumbia' 'Dance music' 'Didgeridoo' 'Ding-dong' 'Disco' 'Double bass'
 'Drone music' 'Drum' 'Drum and bass' 'Drum beat' 'Drum kit' 'Drum machine'
'Drum roll' 'Dub' 'Dubstep' 'Electric guitar' 'Electric piano' 'Electro'
 'Electronic dance music' 'Electronic music' 'Electronic organ'
 'Electronic tuner' 'Electronica' 'Exciting music' 'Female singing'
 'Filing (rasp)' 'Flamenco' 'Folk music' 'French horn' 'Funk'
 'Funk carioca' 'Funny music' 'Glockenspiel' 'Gospel music' 'Gong'
 'Gramophone record' 'Grime music' 'Guitar' 'Guitar amplifier'
 'Hammond organ' 'Happy music' 'Harmonic' 'Harmonica' 'Harmony' 'Harp'
 'Harpsichord' 'Headphones' 'Hi-hat' 'Hip hop music' 'House music'
 'Independent music' 'Jazz' 'Inside, large room or hall'
 'Inside, public space' 'Inside, small room' 'Jingle (music)'
  'Jingle bell' 'Jingle, tinkle' 'Keyboard (musical)' 'Kuduro' 'Kwaito'
'Lullaby' 'MP3' 'Loop' 'Mallet percussion' 'Male singing' 'Mandolin' 'Mantra'
'Maraca'
 'Marimba, xylophone' 'Mellotron' 'Melody' 'Middle Eastern music' 'Music'
 'Music for children' 'Music genre' 'Music mood' 'Music of Africa'
 'Music of Asia' 'Music of Bollywood' 'Music of Latin America'
 'Music role' 'Musical concepts' 'Musical ensemble' 'Musical instrument'
 'Musical note' 'New-age music' 'Noise music' 'Oboe' 'Oldschool jungle'
 'Opera' 'Orchestra' 'Organ' 'Percussion' 'Piano' 'Pizzicato'
 'Plucked string instrument' 'Pop music' 'Progressive rock'
 'Psychedelic rock' 'Radio' 'Rapping' 'Reggae' 'Recording' 'Rhodes piano'
'Rattle' 'Rattle (instrument)' 'Rhythm and blues' 'Rimshot' 'Ringing (of
resonator)' 'Ringtone' 'Rock and roll' 'Rock music' 'Sad music' 'Salsa music'
'Saxophone' 'Scary music' 'Scratching (performance technique)' 'Sidetone'
'Sine wave''Singing' 'Singing bowl' 'Sitar' 'Ska' 'Soca music' 'Song'
 'Soprano saxophone' 'Soul music' 'Soundtrack music' 'String section'
 'Swing music' 'Synthesizer' 'Synthetic singing' 'Tabla' 'Tambourine'
 'Tap' 'Tape hiss' 'Tapping (guitar technique)' 'Techno' 'Tender music'
 'Telephone bell ringing' 'Theme music' 'Theremin' 'Traditional music'
 'Trance music' 'Trap music' 'Trumpet' 'Tubular bells' 'Ukulele'
 'Vibraphone' 'Video game music' 'Violin, fiddle' 'Vocal music'
 'Wedding music' 'Whistle' 'Whistling'
```

'Wind instrument, woodwind instrument' 'Wind noise (microphone)' 'Yodeling' 'Zither']

These lists are present as files in the Huggingface dataset.

After manual listening, we were particularly concerned with the presence of speech sounds, which even after this filtering remained in certain files. Because the speech models showed no emergence, we decided that the model could still be useful. This was reinforced by observations in several other projects showing that there was for this environmental model: no emergence of speech vs unstructured speech categorization (Orhan et al 2025, Phd Thesis), as well as no emergence of music vs unstructured music categorization (Orhan et al. 2025, Phd Thesis). Similarly, no yoking to music or speech structure (i.e adaptation of models' integration windows) was seen in the environmental sound model.
Yet we agree with the reviewer that this environmental sound model could still benefit from music elements here and there in this dataset scrapped from YouTube.
To better quantify the amount of music, we listened precisely to a random subset of the training dataset.
In those 149 files, 9 (6%) of them included music, with 4 of these music tracks blended in the background and made of non-repetitive experimental samples.
12 files (8%) contained clear speech.
In all the remaining files, a large amount: 128 (45%), contained nonmusical sounds but with clear temporal structures.
Examples of sounds with temporal structures include bird chirps, dog barking, tapping sounds, or repetitive mechanic sounds like motors.
Other sounds could contain textured sounds or a seemingly random sequence of unrelated sounds.
Based on this manual verification, we concluded that the contamination of the Audioset dataset with several musical events is neglectable compared to the other natural sounds.

We add in the method section a paragraph dedicated to the pretraining datasets:

The Audioset dataset contains low-quality audio files, with sometimes loose annotations.
We removed from the dataset, all audio files which were annotated with speech or music-related labels.
The exact set of annotations we used is available as CSV files in the HugginFace repository of this dataset (speechlabels.csv and musiclabels.csv).
To verify the quality of the dataset, we then manually listened to a subset of the training dataset, containing 150 files.
In those files, 9 (6\%) of them included music, with 4 of these music tracks blended in the background and made of non-repetitive experimental samples.
12 files (8\%) contained clear speech.
In all the remaining files, a large amount (128 (45\%)) contained nonmusical sounds but with clear temporal structures.
Examples of sounds with temporal structures include bird chirps, dog barking, tapping sounds, or repetitive mechanic sounds like motors.
Other sounds could contain textured sounds or a seemingly random sequence of unrelated sounds.
Based on this manual verification, we concluded that the contamination of the Audioset dataset with several musical events is neglectable compared to the other natural sounds.

To stress the music contamination of the training data, we add the following sentence in the main text:

We first pretrain the model on a general audio dataset, composed of 3x300 hours of public datasets: Librispeech (speech), FMA (music) and Audioset (environmental sounds).
We removed from the Audioset dataset, musical and speech sounds thanks to the accompanying taxonomy. This partially removed all music and speech sounds, with errors (6\% of music and 9\% of speech) remaining due to the poor annotations of the Audioset data.

1.2

2) I am concerned about differences across the 4 tests of algebraic structure being driven by differences in acoustic cues. Short tones vs. syllables could be represented very differently by the neural networks (for instance, in a speech-trained network, tones are out of distribution, and so all tones may have a high loss). To evaluate which parts of the results are driven by low-level cues vs. the higher-order algebraic structure, perhaps the authors could construct two control experiments (1) for chunking that uses tones rather than syllables and (2) for at least one of the other experiments that substitutes syllables in for tones. This would show which differences in Figures 3 and 4 (and similar SI Figs) are due to the underlying audio rather than the actual structure the authors are trying to test.

Author response

To better control that the acoustic similarity played no role in this finding, we extend our tests to a 3x3x4 design , where all **3** models (music, speech, environmental) are tested with all **3** types of sounds (tones, artificial syllables, and, novel, excerpt of environmental sounds) on all **4** protocols. To summarize our results, we report the surprisal to rand metric (similar to Fig 2, C,F,I,L), for each of these conditions. Speech models systematically failed to perform structure detection in all 3x4 conditions, in stark contrast with the music and environmental sounds model. This experiment addresses the same concerns of R1, R2 and R3.

test stimulus	model	AlRoumi2023	Barascud2016	Saffran1996	Bekinschtein2009
natural sound	environmental pretraining	2.50	5.45	4.43	1.58
	music pretraining	7.41	5.88	3.83	6.58
	speech pretraining	**0.16**	**0.48**	**0.02**	**-0.02**
speech	environmental pretraining	0.20	1.96	1.92	0.10
	music pretraining	0.23	2.39	2.26	0.97
	speech pretraining	**-0.04**	**0.17**	**-0.06**	**-0.10**
tones	environmental pretraining	0.76	1.45	1.69	1.68

	music pretraining	4.68	4.50	2.90	4.17
	speech pretraining	**-0.02**	**0.27**	**-0.26**	**-0.19**

Table 2: Cross dataset evaluation: We evaluate each model on the 4 types of sequences changing the sequence elements to environmental sound, syllables, or tones. In all situations, speech models are never able to detect the auditory structure. This shows that the difference between the models is not explained by a difference in the type of test elements.

1.3

3) Claims about training dynamics may be dependent on the choice of learning rates. The learning rate schedule and chosen optimization method for a given model will significantly change the trajectory of training, and different training datasets or models can require different learning rates for end convergence of good representations. How were the learning rates determined for each experiment? Do the results depend on these choices? To make strong claims about differences in training datasets (ie Fig 3) it seems that one would need to show that the trends emerge regardless of optimizer. One way to extend this may be to train the models with a different architecture, as generalization across architectures makes an even stronger claim that this is about the training dataset and not decisions during model pre-training.

Author response:

We agree with the reviewer that learning rate is an important parameter of model optimizations.
Our strongest claim focuses on the absence of emergence in the speech vs the music/environmental sounds models. With respect to this, the learning rate was chosen to be the same for all models, in order to produce a fair comparison. More specifically, as stated in the methods, it was chosen to be the same as in the original paper of Wav2vec2 training. In our hands, changing this learning rate or the optimizer methods leads to gradient explosion in the models, a difficulty that arises in training the Wav2vec2 model, which is not robust to such changes. It was consequently beneficial to our investigation that we could train music or environmental models without the extremely costly exploration of model hyper-parameters. Furthermore, since the models were initially designed for speech processing, this gave a head-start to the speech models. Despite this advantage, the speech models did not show an emergence of a structure detection algorithm.
To strengthen our conclusions, we consequently searched for change in model architecture that would still allows pretraining with minimal hyperparameters exploration.
During our exploration, we observed that one type of modification that would not prevent model training was to diminish the number of layers. To question if our results were robust to these choices, we evaluated 2 tiny versions of Wav2vec2, that used only 3 transformer layers.
One version of the model was trained on speech and the other on the music dataset. Our results were unchanged: tiny speech models could not detect auditory regularities in contrary to the tiny music model.

We add to the main text the following sentence:

> Next, we questioned if these results were robust to change in models' architecture.

One architecture change that would not disturb model pretraining was to reduce the number of layers in the model. We trained tiny music and speech models with 3 instead of 12 transformer layers, our results were robust to these changes.

model type	model	AlRoumi2023	Barascud2016	Saffran1996	Bekinschtein2009
wav2vec2 tiny	music tiny	4.16	3.22	2.72	2.96
	speech tiny	**-1.17**	**0.06**	**-0.44**	**-0.58**
wav2vec2	music base	4.68	4.50	2.90	4.17
	speech base	**-0.02**	**0.27**	**-0.26**	**-0.19**

Table 3. Differences between music and speech models are robust to a change in the model architecture.

Minor Comments:

1.4

- Methods: I could not find the exact amount of unique data included for each of the sound sets (I guess this is ~300 hours of each set, according to line 101, but I found this description confusing). The axes are related to the hours of pretraining, but it is important to know how many passes through unique examples are included in each experiment. It might be helpful to include this in some way for the main text figures.

Author response:

We now include an extensive table with the detailed amount of time included in each dataset. We also clarified the line 101.

	Librispeech	FMA	Audioset
Number of hours	951	587.9	622.1
Number of audio files	277710	106395	227700

Table 1. Comparison of audio datasets in terms of duration and number of files.

1.5

- General Wording: The abstract and introduction contain some jargon that is not well defined and may not be common to some more general readers. For instance “merge” and “neural recursion” are a bit distracting at the start of the paper without giving the readers a little more context.

Author response:

We removed the reference to “merge” and “neural recursion” from the abstract.

1.6

- Citations: There is quite a bit of work in the Computational Cognitive Neuroscience world that touches on how differences in training environments shape the behavior and internal representations of neural networks. Although much of this work focuses on supervised

networks, the general claims about training environments seem relevant to this work. Some examples here, but I encourage the authors to do a further literature review:
https://www.nature.com/articles/s41562-021-01244-z
https://journals.plos.org/plosbiology/article?id=10.1371/journal.pbio.3002366
https://www.nature.com/articles/s41467-024-53147-y
https://www.nature.com/articles/s41467-023-44516-0

Author response:
We add the following references to the discussion:

Alternatively to these self-supervised emergences, previous works have measured how the training environment impacts supervised model processes. Notably, Francl et al. revealed that human-like sound localization only emerged in environments including reverberation, noise, or natural sounds. Tuckute et al. stressed the importance of training the model in noisy speech environments to better predict brain responses to speech. The common denominator of these works is to illustrate the importance of approaching a realistic developmental environment to better model brain and behavioral responses. Closer to our work, Kim et al. measured the existence of units responsive to music shuffling at sizes up to 400 ms in convolution neural networks trained to classify environmental sounds.

Typos:
-Line 57: “critical to build system” -> “critical to build systems”
-Line 162: ‘ is facing the wrong way for Repeating tones
-Line 382: Some words are missing or out of order in the first part of this sentence.

Author response:
We thank the reviewer for spotting these typos.
